# Coupling and Activation of the β1 Adrenergic Receptor - The Role of the Third Intracellular Loop

**DOI:** 10.1021/jacs.4c11250

**Published:** 2024-10-03

**Authors:** Xingyu Qiu, Kin Chao, Siyuan Song, Yi-Quan Wang, Yi-An Chen, Sarah L. Rouse, Hsin-Yung Yen, Carol V. Robinson

**Affiliations:** † Physical and Theoretical Chemistry Laboratory, Department of Chemistry, University of Oxford, Oxford, OX1 3QZ, U.K.; ‡ Kavli Institute for Nanoscience Discovery, Dorothy Crowfoot Hodgkin Building, University of Oxford, Oxford, OX1 3QU, U.K.; § Department of Life Sciences, Imperial College London, South Kensington Campus, London, SW7 2AZ, U.K.; ∥ Institute of Biological Chemistry, Academia Sinica, Taipei, 115024, Taiwan

## Abstract

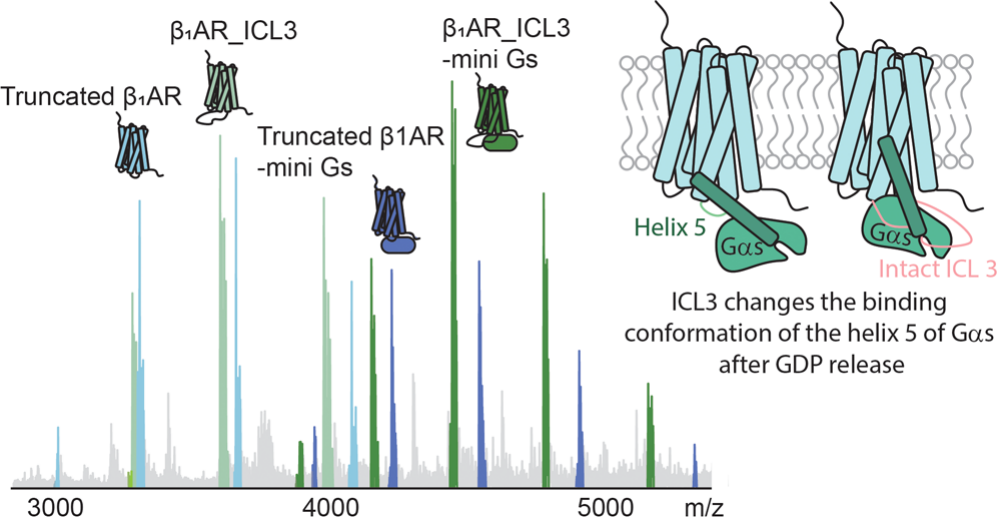

G protein-coupled receptors (GPCRs) belong to the most diverse group of membrane receptors with a conserved structure of seven transmembrane (TM) α-helices connected by intracellular and extracellular loops. Intracellular loop 3 (ICL3) connects TM5 and TM6, the two helices shown to play significant roles in receptor activation. Herein, we investigate the activation and signaling of the β_1_ adrenergic receptor (β_1_AR) using mass spectrometry (MS) with a particular focus on the ICL3 loop. First, using native MS, we measure the extent of receptor coupling to an engineered Gα_s_ subunit (mini G_s_) and show preferential coupling to β_1_AR with an intact ICL3 (β_1_AR_ICL3) compared to the truncated β_1_AR. Next, using hydrogen–deuterium exchange (HDX)-MS, we show how helix 5 of mini G_s_ reports on the extent of receptor activation in the presence of a range of agonists. Then, exploring a range of solution conditions and using comparative HDX, we note additional HDX protection when ICL3 is present, implying that mini G_s_ helix 5 presents a different binding conformation to the surface of β_1_AR_ICL3, a conclusion supported by MD simulation. Considering when this conformatonal change occurs we used time-resolved HDX and employed two functional assays to measure GDP release and cAMP production, with and without ICL3. We found that ICL3 exerts its effect on G_s_ through enhanced cAMP production but does not affect GDP release. Together, our study uncovers potential roles of ICL3 in fine-tuning GPCR activation through subtle changes in the binding pose of helix 5, only after nucleotide release from G_s_.

## Introduction

G protein-coupled receptors (GPCRs) constitute the largest families of transmembrane proteins, with around 800 receptors classified into five major classes based on their structural features. The molecular basis of GPCR activation and modulation is intricate, owing to their highly diverse structural features for ligand recognition and the convergence of their G protein-coupling. GPCRs share a similar architecture of seven-transmembrane domains with loops connecting the transmembrane helices on both sides of the lipid bilayer. These loops are thought to play essential roles in ligand binding to the receptor.^1,2^ The intracellular loop 3 (ICL3) connects TM5 and TM6, the two helices known to participate in G protein coupling.

Upon agonist stimulation, TM5 and TM6 of a GPCR undergo significant conformational changes to create a binding cavity for the recruitment of G proteins.^1,2^ Several lines of evidence suggest that participation of ICL3 in this interaction influences the activation pathway of the receptor.^3−11^ For example, previous research has indicated that ICL3 of the β_2_ adrenergic receptor (β_2_AR) plays a role in conferring the coupling selectivity of the G_s_ protein.^4,5^ Substitution of the C-terminal parts of β_2_AR ICL3, by the sequence of the α_2_ Adrenergic receptor (α_2_ AR) ICL3, drastically weakened the coupling of β_2_AR to G_s_.^4−6^ Using an assay based on accumulation of second messengers (cAMP and InsP_1_), a recent study also revealed that absence of ICL3 promotes GPCR recruitment of noncognate G proteins.^11^ Additionally, peptides from the ICL3 of both the α_2_AR and the Dopamine D2 receptor demonstrated a strong affinity to G_o_ and G_i_.^11^ These synthetic peptides were also shown to facilitate GTPase activity of G_i/o_ implying a possible role for ICL3 in G_i/o_ activation.^3,7−10^ Moreover, ICL3 was found to participate in the binding of GPCRs to β-arrestin.^12−17^ Together, these different observations indicate that ICL3 has unique functions in both the canonical and noncanonical signaling pathways of GPCRs. However, due to its flexibility, ICL3 is usually removed in structural studies or sometimes replaced by fusion proteins such as T4L lysozyme and BRIL for thermo-stabilization purposes. As intact ICL3 of β_1_AR has not been observed in previous high-resolution structural studies,^18^ the impact of this loop on coupling is not fully understood.

**Figure 1:**
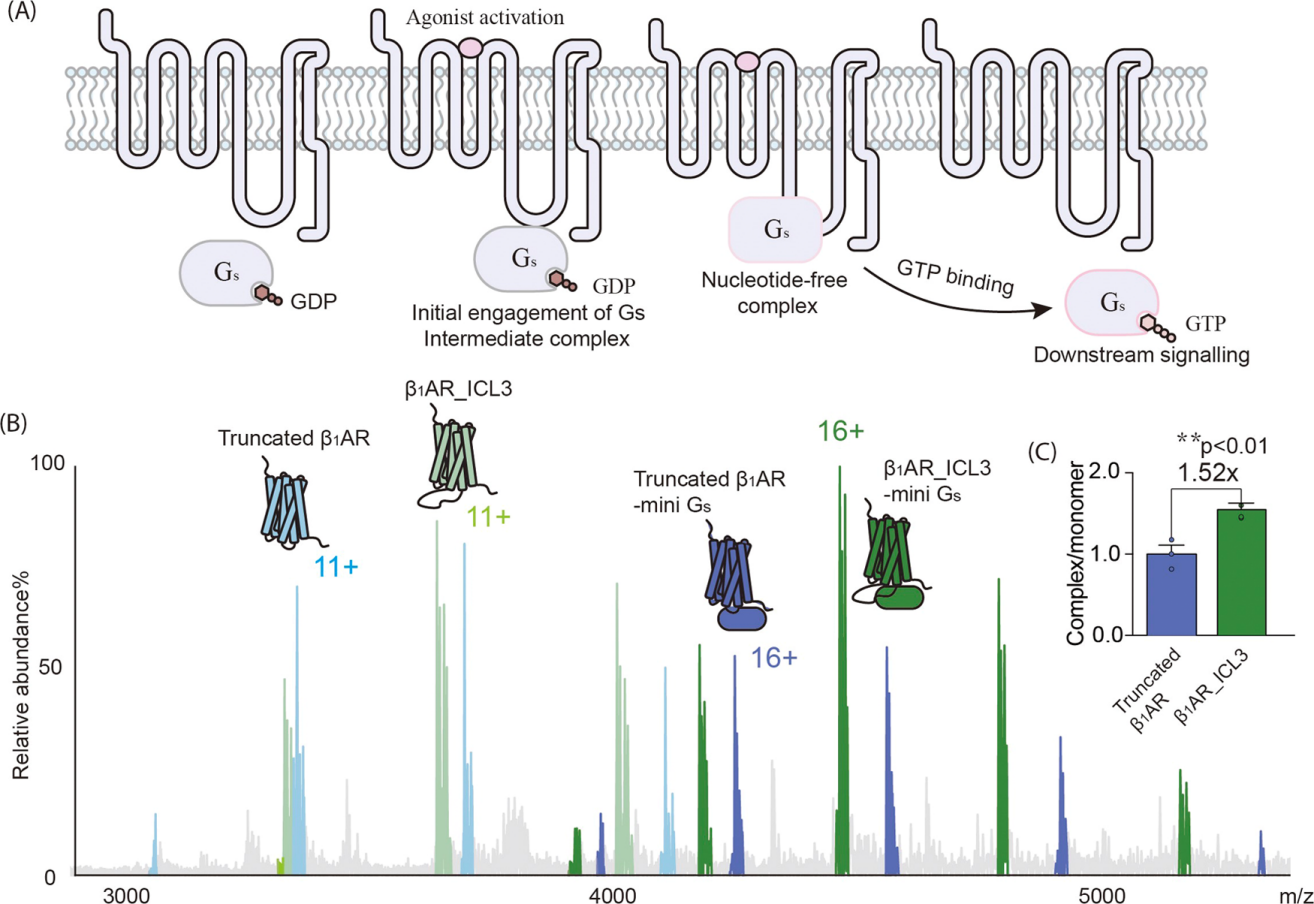
Native MS analysis of β_1_AR (Truncated β_1_AR and β_1_AR_ICL3) in complex with mini G_s_. (A) Schematic of the G protein signaling process. An agonist binds to the extracellular side of the GPCR to induce the initial engagement of the receptor to the G protein. This results in the formation of an intermediate complex and a conformational change of G_s_ then mediates GDP release. GTP binding to the nucleotide free complex forms the active G protein (G_s_.GTP) which then dissociates from the complex for downstream signaling. (B) Peaks in the mass spectrum are assigned to β_1_AR constructs and their complexes coupled to mini G_s_ after incubation for 20 min in the presence of isoprenaline (5-fold excess). Apo truncated β_1_AR, apo β_1_AR_ICL3, colored pale blue and green respectively, are incubated with an equivalent conconcentration of mini G_s_ (all at 2.5 μM) to establish between the receptor constructs for mini G_s_. Truncated β_1_AR-mini G_s_ and β_1_AR_ICL3 mini G_s_ complexes are colored darker blue and green, respectively. The complex:monomer ratio of β_1_AR_ICL3 is significantly higher than the ratio of truncated β_1_AR-mini G_s_:truncated β_1_AR (bar chart (C) upper right). Error bars are generated according to the standard deviation calculated from three independent repeats. A student’s *t*-test was used to evaluate the statistically significant differences (*p* < 0.01).

Considering where ICL3 might exert an effect in the established steps of GPCR signaling, which starts with engagement of helix 5 from the G protein with the GPCR,^19^ the receptor then induces a conformational change of the G protein to release GDP (Figure 1A). The G protein then binds to GTP and dissociates from the receptor for downstream signaling and cyclic AMP (cAMP) production. In the presence of ligands, previous structural studies of β_2_AR presented evidence for initial engagement of the receptor with a GDP-bound G protein, or an intermediate complex.^19−22^ To our knowledge, the impact of ICL3 on this intermediate complex has not been investigated.

We set out to define the effects of ICL3 on G protein coupling on the formation and stability of the intermediate complex and on cAMP production using a combination of native MS and HDX. Both MS approaches have been employed previously for GPCRs, quantifying the basal activity of β_1_AR and the conformational changes of various GPCRs.^19,23,24^ Here, we apply these approaches to reveal the extent of β_1_AR coupling to mini G_s_ (the engineered GTPase domain of the G_αs_ protein^25^). First in the presence of different agonists and then with and without ICL3, HDX-MS reveals the extent of G-protein engagement. Subsequently by varying the concentration of the receptors for HDX-MS, and performing molecular dynamics (MD) simulations, we show that upon coupling ICL3 can change the binding pose of helix 5 of mini G_s_. By performing a cAMP accumulation assay, we then found the promotional effect of ICL3 on signal transduction of β_1_AR. We also applied a GDP binding assay, time-resolved HDX, and native MS, to demonstrate that ICL3 of β_1_AR changes the coupling mode of G_s_ helix 5, downstream of the intermediate complex, specifically at the nucleotide-free state of the complex.

## Results and Discussion

### ICL3 promotes β_1_AR coupling to mini G_s_

To investigate the influence of ICL3 on GPCR-G protein coupling, we recorded native mass spectra of the two receptor constructs (described below) with and without ICL3, and mini G_s_ (Figure 1B). Mini G_s_ has been reported to form stable complexes with several GPCRs, including the adenosine A_2A_ receptor (A_2A_R) and β_1_AR, and to increase the agonist affinity of receptors allosterically.^25^ The structure of the A_2A_R coupled to mini G_s_ revealed similar activation features observed in the β_2_AR–G_s_ complex such as outward movement of TM6 and interaction between helix 5 of G_s_ with the intracellular side of the receptor.^26,27^

We selected β_1_AR for our investigation expressing the construct for truncated β_1_AR (β_1_AR without an intact ICL3) from *M. gallopavo* β114-E130W (β_1_AR), based on the previously published thermostabilized β_1_AR44-m23 construct.^28,29^ Compared to the β_1_AR44-m23, our construct (β114-E130W) omits two point mutations at TM5 and TM6 (Y227A and A282L), which allow β114 to couple to G proteins in the presence of an agonist.^25^ The additional mutation, E130W of β_1_AR, promotes functional expression of β_1_AR as well as allows for the receptor to be purified without a bound ligand. For β_1_AR with an intact ICL3 (defined here as β_1_AR_ICL3) we used the *M. gallopavo* β114E130WIC3 where the ICL3 sequence was added back in to β114-E130W (Supplementary Figure 1). The mass spectrometry coupling experiments are based on a method described previously.^30^ Briefly, truncated β_1_AR is incubated with β_1_AR_ICL3 at a 1:1 molar ratio (2.5 μM) with mini G_s_ (2.5 μM) and a 5-fold excess of isoprenaline for 20 min. Native mass spectra are then recorded under conditions such that both apo β_1_AR and β_1_AR_ICL3 are detected (Figure 1B). Results show that the peaks assigned to the β_1_AR_ICL3 mini G_s_ complex are significantly more intense than those observed for the truncated β_1_AR-mini G_s_ complex. (Peak intensities of the monomers and complexes were quantified by using UniDec software.) We compared the ratio of the peak intensities assigned to the complex: apo receptor for the two receptors (Figure 1C). Since we are comparing ratios rather than absolute peak intensities, changes in ionization efficiency can be discounted. The significantly higher intensity ratio for β_1_AR_ICL3:β_1_AR_ICL3 mini G_s_ compared to truncated β_1_AR:truncated β_1_AR-mini G_s_ implies that ICL3 promotes the coupling of β_1_AR to mini G_s_. The next step is to understand how this enhanced coupling is achieved.

### Regions of mini G_s_ helix 5 involved in the activation of β_1_AR

We conducted HDX-MS of the receptor and mini G_s_ to locate regions of the G protein involved in coupling. First we identified multiple peptides of mini G_s_ following pepsin digestion and obtained a sequence coverage of 91% (Supplementary Figure 2). We then considered the concentration of mini G_s_ needed for full-complexation of the receptor in solution. Previous experiments revealed specific binding of mini G_s_ to β_1_AR with an apparent *K*_D_ value of 200 nM.^31^ Recording native MS we observed full complex formation, in the absence of ICL3, at a receptor concentration of 1.5 μM, 1.2 equiv of mini G_s_, consistent with the high affinity of this construct (Supplementary Figure 3). We therefore used this ratio of receptor to mini G_s_ (1.2:1) to conduct HDX-MS analysis.

**Figure 2:**
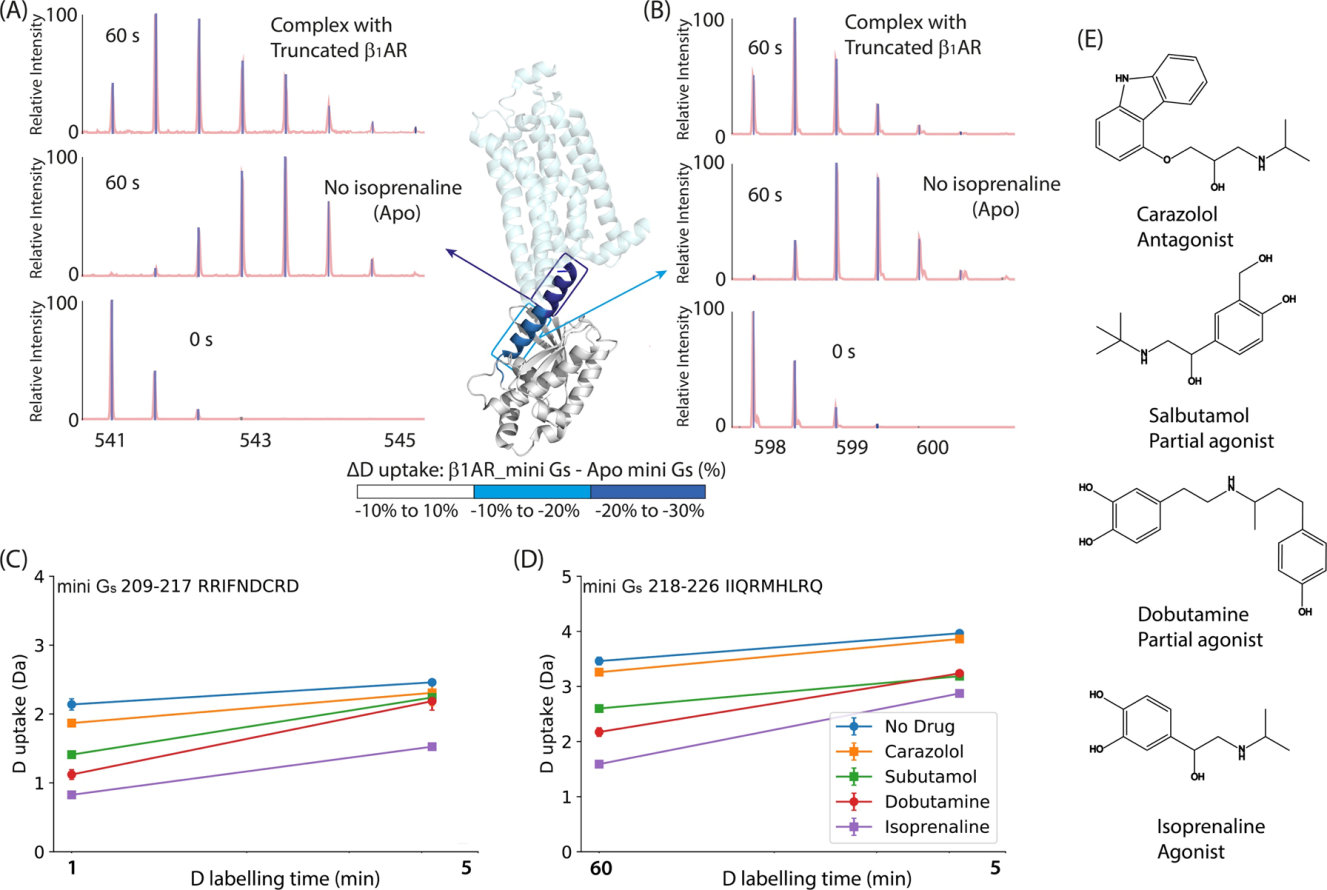
HDX protection of helix 5 in mini G_s_ reports on the activity of truncated β_1_AR in the presence of agonists. (A–B) Deuterium uptake spectra of peptides (A) “IQRMHLRQ” and (B) “RRIFNDCRD” from mini G_s_ helix 5 in complex with β_1_AR (without ICL3) with and without isoprenaline, after 60 s labeling time and an incubation time of 20 min. Heat maps of the degree of HDX difference between apo mini G_s_ and mini G_s_ in complex with truncated β_1_AR (‘HDX percent β_1_AR-mini G_s_ - HDX percent of apo β_1_AR-mini G_s_). Regions are highlighted based on the structure of mini G_s_ coupled to a class A receptor (A_2A_R), for illustrative purposes.^27^ (C–D) Deuterium uptake is plotted as a function of labeling time in the presence of four drugs and compared to the apo form of the receptor. (C) 209–217 assigned to the N-terminal residues of mini G_s_ helix 5. (D) 218–226 assigned to the C-terminal residues of mini G_s_ helix 5. Error bars are generated according to the standard deviation calculated from three independent repeats. A student’s *t* test was used to evaluate the statistically significant differences (*p* < 0.01). (E) Structures of the four drugs used here.

Next we compared the HDX properties of mini G_s_ incubated with apo β_1_AR and activated with isoprenaline (coupling method as reported^30^ and reproduced in the Supporting Information). Following complex formantion (incubation time 20 min with 60 s of labeling) a significant reduction of deuterium uptake in the presence of isoprenaline was observed, compared to the apo form of β_1_AR without isoprenaline. On average a reduction of 20–30% and 10–20% was observed for the C- and N-terminal peptides respectively of helix 5 (Figure 2A–B). This observation suggests that after incubation both the N and C terminal ends of helix 5 of mini G_s_ are strongly protected, the effect being more pronounced for the C-terminus (residues 219–216 IQRMHLRQ) given its greater burial in the hydrophobic core of the receptor. We next explored three further deuterium labeling times (10, 250, and 1000 s) using the same incubation time. Under all conditions we observed protection of helix 5, the effect reducing with longer labeling times (Supplementary Figure 4). These results are together consistent with the insertion of helix 5 into the hydrophobic transmembrane region of the receptor upon complex formation.

As a control experiment, we also conducted HDX-MS analysis on mini G_s/i_ since β_1_AR couples to G_s_.^32^ (Mini G_s/i_ is a chimeric protein with helix 5 of G_i_.) After incubation for 20 min of mini G_s/i_ with isoprenaline-bound β_1_AR, and after labeling for 60 s, no deuterium uptake change was observed for helix 5 of mini G_s/i_ (Supplementary Figure 5). Since the structure of β_1_AR coupled to G_i_^33^ shows that helix 5 of G_i_ interacts differently with the receptor, our HDX-MS result, which does not capture the interaction of helix 5 of mini G_i_ with β_1_AR, does not rule out a weak interaction in a distinct conformation (Figure 2A-B). Nevertheless, our results suggest that HDX-MS could potentially reveal the extent and selectivity of β_1_AR coupling to mini G_s_. If this is the case, then we hypothesize that a series of ligands with different agonist effects would lead to differences in HDX protection because of the extent of their coupling.

We tested three drugs, dobutamine, salbutamol, and carazolol, in addition to isoprenaline for their ability to induce HDX protection in helix 5 of mini G_s_. The three additional drugs we selected are (i) Carazolol, a nonselective antagonist to both β_1_ and β_2_ adrenoreceptors;^34^ (ii) Dobutamine, a partial agonist of β_1_AR^35^ used for the treatment of cardiogenic shock; and (iii) Salbutamol, a partial agonist of β_2_AR,^36^ used to relieve symptoms of asthma by relaxing smooth muscle in the airway. We added the drugs individually to a solution of β_1_AR and mini G_s_ and recorded the HDX protection focusing on helix 5 of mini G_s_ (Figure 2C–D). Comparing the drugs to the solution of apo β_1_AR with mini G_s_ we found that for carazolol (antagonist), there is no difference in protection of helix 5. By contrast, isoprenaline-bound β_1_AR yielded the strongest protection, while β_1_AR bound to dobutamine or salbutamol conferred less protection. These HDX results suggest that the conformational state of helix 5 of mini G_s_ is not only a sensitive reporter of complex engagement but also serves as a proxy for the activity of β_1_AR (Supplementary Figure 5C).

### ICL3 influences the coupling of mini G_s_ helix 5

**Figure 3:**
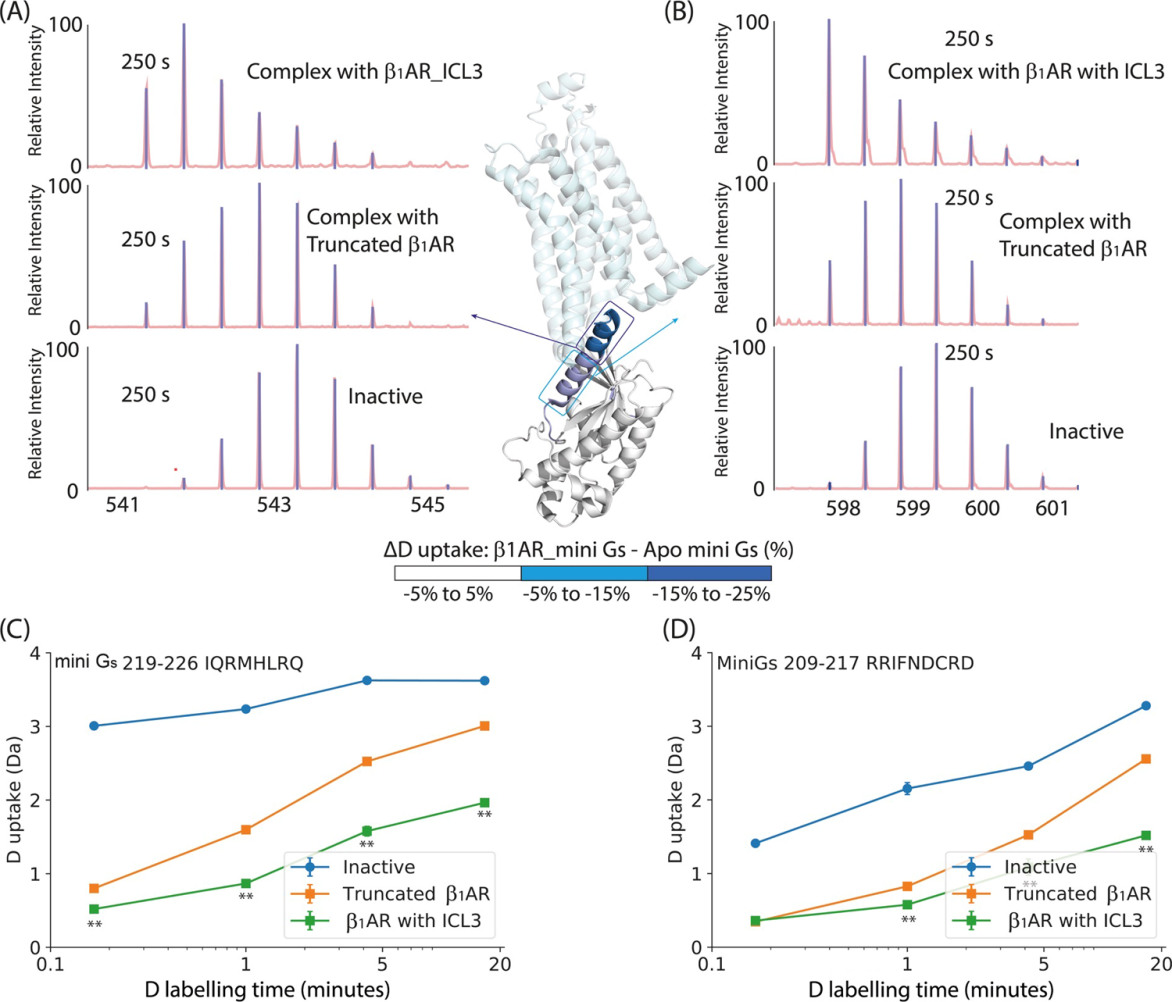
ICL3 increases HDX protection of G_s_ helix 5 upon β_**1**_AR coupling with truncated and β_**1**_AR_ICL3. (A–B) Deuterium uptake spectra of mini G_s_ helix 5 peptides (A) “IQRMHLRQ” C-ter and (B) “RRIFNDCRD” N-ter in complex with β_1_AR _ICL3 or the truncated construct (both activated and in 1.2 equiv excess) and the inactive truncated β_1_AR. Deuterium uptake is plotted as a function of labeling time (250 s) and shown for (A) the C-terminus and (B) the N-terminus of G_s_ helix 5. (C–D) D uptake is plotted as a function of deuterium labeling time from 10 to 1000 s for these two peptides. Error bars are generated according to the standard deviation calculated from three independent repeats. A student’s *t* test was used to evaluate the statistically significant differences (*p* < 0.05*, *p* < 0.01**). Heat maps of the HDX fractional uptake difference between the truncated β_1_AR and β_1_AR _ICL3 complexes at a deuterium labeling time of 250 s (i.e., [% HDX mini G_s_-β_1_AR_ICL3] – [% HDX mini G_s_-β_1_AR_truncated]). Differences are displayed on the structure of mini G_s_ coupled to A_2A_R, for illustrative purposes.^27^

To examine the effects of ICL3 on coupling, we used the HDX-MS approach described above. We compared (i) the active state of β_1_AR_ICL3 + isoprenaline, (ii) the active state of truncated β_1_AR + isoprenaline, and (iii) inactive β_1_AR (− isoprenaline). All three were incubated individually with mini G_s_ for 20 min. Comparing N- and C-terminal helix 5 peptides from the three experimental conditions we observe strikingly greater protection in the active β_1_AR_ICL3 compared to β_1_AR (absence of ICL3) and the inactive states (− isoprenaline) (Figure 3 and Supplementary Figure 5D-E). Comparing both activated complexes, mini G_s_–β_1_AR_ICL3 and mini G_s_-β_1_AR complex, shows that ICL3 facilitates further protection of helix 5 over that conferred to the mini G_s_-β_1_AR complex. Both activated complexes, in turn, exhibit greater protection than the intactive one. This observation is in line with our proposal above that HDX protection of helix 5 is a reporter for the activation of β_1_AR (Figure 2C–D) and demonstrates that ICL3 promotes further the activity of β_1_AR.

**Figure 4:**
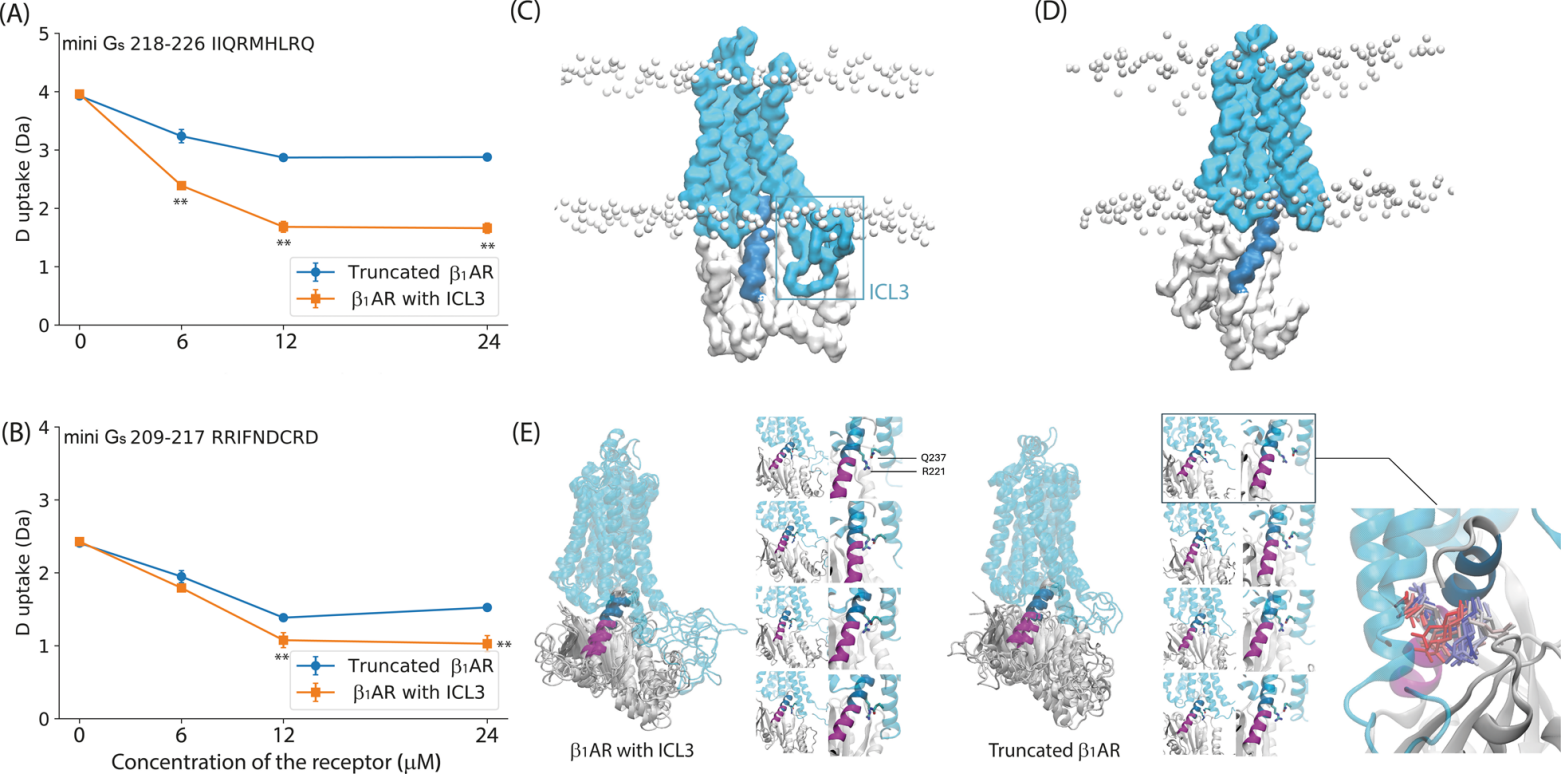
Impact of ICL3 on HDX protection of mini G_s_ helix 5 and the binding orientation of mini G_s_. (A–B) Concentration-dependent HDX-MS measured only for mini G_s_ helix 5 in complex with β_1_AR_ICL3 or the truncated construct (deuterium labeling time: 250 s). The concentration of mini G_s_ is fixed at 20 μM and deuterium uptake is plotted as a function of receptor concentration (from 0 to 24 μM) for two selected mini G_s_ helix 5 peptides: (A) the C terminal residues and (B) the N terminal residues. The error bars are generated according to the standard deviation calculated from three independent repeats. A student’s *t*-test was used to evaluate the statistically significant differences (*p* < 0.01). (C–D) Coarse-grained molecular dynamics simulations: In the presence of ICL3, helix 5 of mini G_s_ tends to adopt a more closed binding orientation (C), compared to the orientation preferred by truncated β_1_AR (D). (E) The final frames of 4 × 2 μs atomistic molecular dynamics simulations. The left-hand panel shows the hydrogen bond between R221 of mini G_s_ helix 5 and Q237 of β1AR for β1AR_ICL3, where 3 out of 4 repeats maintained a hydrogen bond with the terminal charged NηH_2_^+^ in R221 and 1 repeat with the weaker NεH in R221. The right-hand panel shows the hydrogen bond for truncated β_1_AR, where 3 out of 4 repeats formed hydrogen bonds with the weaker NεH in R221, and in one repeat, the hydrogen bond is completely broken, allowing a more prounced change in orientation of mini G_s_. The zoomed-in view (rhs) shows the time series of the hydrogen bond breakage in the truncated β_1_AR (red to blue: initial to final frames shown at 200 ns intervals).

To explore further the origin of this additional HDX protection in helix 5, and how it might change as equilibrium is perturbed, we manipulated the concentration of the receptor (0 μM to 24 μM), fixed the concentration of mini G_s_ (20 μM) and isoprenaline (100 μM), and the labeling time (250 s). The deuterium uptake of both the N-terminal and C-terminal peptides of mini G_s_ helix 5 decreases as the receptor concentration increases. In other words, protection increases as the concentration of the receptors increases (Figure 4A-B). In both cases, for the truncated β_1_AR and β_1_AR_ICL3, 0.6 equiv of receptor was sufficient to reach saturation of protection for helix 5. However, the extent of protection to the C-terminus of helix 5 was greater for β_1_AR_ICL3 than for β_1_AR at all concentrations tested. Together, these HDX results support our observations from native MS wherein mini G_s_ preferentially couples to β_1_AR_ICL3. We suggest therefore that the enhanced protection observed in the presence of ICL3 must arise from a conformational change of mini G_s_ helix 5 during complex formation, which contributes to its increased binding affinity.

### MD reveals differences in mini G_s_ during coupling

To obtain structural insights into the effect of ICL3 on the orientation of mini G_s_, we carried out coarse grained molecular dynamics (cgMD) simulations for the β_1_AR-mini G_s_ complex with and without ICL3. (MD modeling is based on the *M. gallopavo* β_1_AR cryo EM structure of dobutamine−β_1_-AR–Gs (8DCR)^37^Supplementary Figure 6A-B.) During cgMD simulations of the complex, in a model plasma membrane, two distinct orientations of mini G_s_, relative to truncated β_1_AR, were observed: one in which the initial cryo-EM orientation was maintained (Supplementary Figure 6C), and another in which mini G_s_ is shifted such that it no longer packs against TM5 and TM6 of β_1_AR (Supplementary Figure 6D). We then compared these truncated structures with those in the presence of ICL3 (highlighted in Figure 4C). Over the course of the 5 μs simulation, the angle formed by the orientation of helix 5 of mini G_s_, to the plane by three residues in β_1_AR_ICL3, is consistently lower than that for the same residues in β_1_AR (Supplementary Figure 6E). Representative structures show that β_1_AR_ICL3 couples in a more “closed” orientation than truncated β_1_AR in all 5 cgMD replicates (Figure 4D). Specifically, ICL3 was observed to pack against mini G_s_ as well as to interact with the model plasma membrane. In the absence of ICL3 mini G_s_ adopted a shifted, “open” orientation in which helix 5 of mini G_s_ is exposed to solvent. Furthermore, our atomistic MD simulation shows that ICL3 enhances a hydrogen bond interaction between the terminal charged NηH_2_^+^ in R221, equivalent to R385 of wild-type G_s_, on helix 5 of mini G_s_ and Q237 of β_1_AR (Figure 4E, Supplemenary Figure 7B-E). In contrast, in the case of truncated β1AR, a weaker hydrogen bond interaction with NεH in R221 was observed, and in one repeat, the complete breakage of the hydrogen bond was seen, resulting in a more shifted mini G_s_ state. Together, our MD results are in line with our HDX-MS study, which indicates that β_1_AR_ICL3 yields a more protected patch on helix 5 of mini G_s_, specifically at the sequence encompassing R221 from I_219_ to Q_226_ (IQRMHLRQ).

### ICL3 of β_1_AR promotes coupling after GDP release

Both the native MS and HDX-MS experiments reported above captured the nucleotide-free state of mini G_s_ in complex with β_1_AR, after 20 min of incubating the receptor and mini G_s_ protein. To investigate the possible influence of ICL3 on proposed intermediate states of coupling, i.e., to the GDP-bound mini G_s_, we need to explore earlier time points in the coupling reaction. We therefore performed time-resolved HDX-MS (or pulsed HDX). We fixed the deuterium labeling time at 10 s and varied the incubation time of activated receptor and mini G_s_ from 10 s to 1 h at 20 °C. A short deuterium labeling time (10 s) should minimize additional coupling taking place during the labeling period. Under these conditions, we found that ICL3 did not induce significant protection on helix 5 of mini G_s_ compared to the construct without ICL3 at the first two coupling incubation times (10 s and 1 min) (Supplementary Figure 8). After 1 h of coupling incubation, a small extent of protection induced by ICL3 was captured. We surmise that a labeling time of 10 s is too short and the HDX differences too subtle for us to draw conclusions.

**Figure 5:**
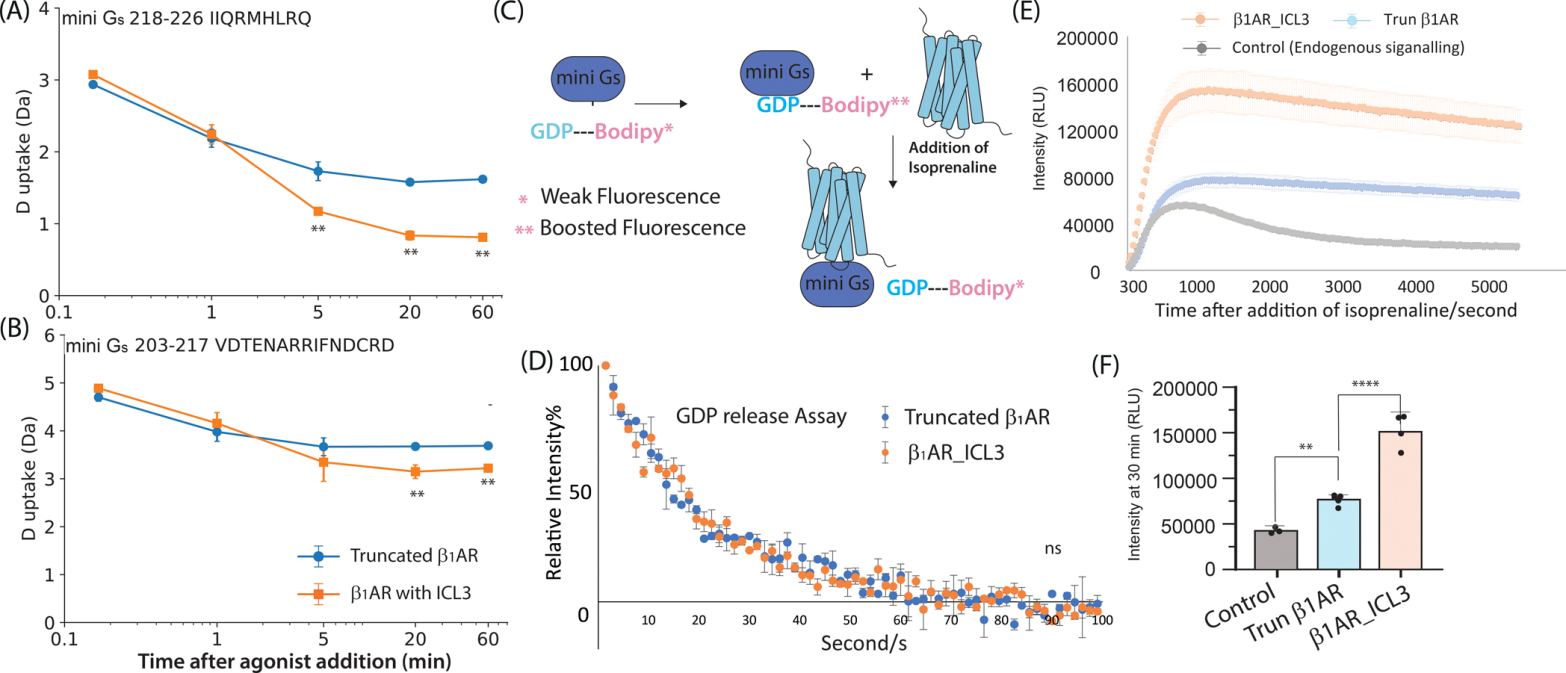
The effect of ICL3 on β_1_AR coupling to mini G_s_ occurs postnucleotide-release. (A–B) Time-resolved HDX-MS of helix 5 in complex with β_1_AR_ICL3 or the truncated construct without ICL3 (Deuterium labeling time: 60 s). Deuterium uptake is plotted as a function of coupling incubation time (time after agonist addition) from 10 s to 1 h for 2 selected peptides covering (A) the C terminus and (B) the N terminus of mini G_s_ helix 5. Error bars are generated according to the standard deviation calculated from three independent repeats. A student’s *t*-test was used to evaluate the statistically significant differences (*p* < 0.01). (C) Schematic to show how BODIPY FL GDP can be used to monitor the release of GDP from the GPCR-mini G_s_ complex. (D) GDP release assay for truncated β_1_AR (Blue) and β_1_AR_ICL3 (Orange). The Relative fluorescence intensity (FL intensity) % is plotted as a function of time upon isoprenaline activation. The value is calculated from (Recorded FL intensity – Basal FL intensity)/(Highest FL intensity – Basal FL intensity) × 100%. Recorded FL intensity represents the average FL intensity (A.U) recorded in a 15 ms period. Basal FL intensity represents the average fluorescence intensity at the final 300 scans. The highest FL intensity stands for the average fluorescence intensity during the initial 15 scans (0–15 ms). Error bars are generated according to the standard deviation calculated from three independent repeats. (E–F) cAMP accumulation assay for HEK293 with overexpressed β_1_AR_ICL3, Truncated β_1_AR, and empty HEK293 as a control (gray) shows that ICL3 promotes the canonical signaling of β_1_AR. (E) Glosensor Luminescence (RLU) of control (gray), Trun β_1_AR (blue) and β_1_AR_ICL3 (orange) is plotted against the time after addition of isoprenaline (full agonist) to HEK293T. (F) Glosensor Luminescence (RLU) of β_1_AR_ICL3, Trun β_1_AR, and control after 30 min incubation with isoprenaline is plotted as bar chart. Error bars are generated according to the standard deviation calculated from three independent biological repeats (control), four independent biological repeats (β_1_AR_ICL3), and five independent biological repeats (Truncated β_1_AR). A one-way ANOVA test was used to evaluate the statistically significant differences (*p* < 0.01). ICL3 was found to significantly promote G_s_ signaling of β_1_AR.

We therefore repeated the experiment above but this time using a longer labeling time of 60 s. Under these conditions we found that helix 5 of mini G_s_ increased in protection gradually as the coupling incubation time increased from 10 s to 20 min at 20 °C, reaching a saturation level after 20 min (Figure 5A-B). This observation implies that we can capture discrete stages of β_1_AR complex formation on a time scale of several minutes. Surprisingly, we observed that the deuterium uptake of helix 5 when coupled to truncated β_1_AR or β_1_AR_ICL3 showed no difference at the initial stages of complex formation (10 and 60 s). Differences in helix 5, but no other regions of mini G_s_, only become apparent after 5 min of coupling and become saturated at 20 min. We align these results with our native MS analysis, wherein coupling is fully realized with no GDP binding detected (incubation time 20 min at 20 °C) (Supplementary Figure 3) and note that the extent of coupling does not change but HDX protection increases gradually. Collectively therefore our data suggest that ICL3 induces a protected patch on helix 5 after the initial stages of complex formation (>1 min) in the nucleotide-free β_1_AR-mini G_s_ complex.

To explore further the influence of ICL3 on GDP release, we set up an orthogonal fluorescence assay to explore the time scale of GDP release during β_1_AR-mini G_s_ coupling. We compared the GDP release rate triggered by truncated β_1_AR with that of β_1_AR_ICL3 using BODIPY FL GDP, a modified GDP analogue with a Bodipy dye connected to the 2′ position of the ribose ring via an aminoethylcarbamoyl linker. The fluorescence of Bodipy dye is suppressed by purine (in GDP) via electron transfer quenching in solution. When the analogue binds to a GTPase, such as mini G_s_, the interaction between the G Protein and the purine reduces the efficiency of electron transfer quenching, leading to the promotion of fluorescence. Upon the addition of an active GPCR, the release of GDP, triggered by the receptor coupling event, results in a decrease in the fluorescence (Figure 5C). Thus, GDP release can be captured by monitoring changes in fluorescence intensity.

To conduct the GDP release assay, an excess of β_1_AR (either truncated or with ICL3) was incubated with mini G_s_ and BODIPY FL GDP in a 96 well plate prior to the fluorescence assay (see Supporting Information for detailed conditions). Immediately after the addition of isoprenaline, fluorescence intensity was monitored over time at 20 °C. GDP release during coupling was successfully captured via a decay in fluorescence as a function of time, decreasing until around 60 s (Figure 5D). This observation implies that GDP release is complete within ∼60 s at 20 °C. A Kolmogorov–Smirnov test for the two distributions showed that the *p* value was larger than 0.95. Importantly, the GDP release curves for truncated β_1_AR and the β_1_AR_ICL3 follow the same distribution implying that the initial stages of GDP release are not impacted by the presence or absence of ICL3.

To achieve full coupling of mini G_s_ to β_1_AR, we used a mini G_s_ mutant (I208A) throughout our study. This mutation not only enhances the affinity of mini G_s_ for β_1_AR but also makes the complexes resistant to GTP-induced dissociation. Given that this mutation could potentially affect our GDP release assay, as a control, we also performed a GDP release assay using mini G_s_ without the alanine mutation at I208 (mini G_s_ I208). First we considered the affinity of the two β_1_AR constructs for mini G_s_ I208 via native MS (Supplementary Figure 9A-B). The coupling experiments were conducted separately for the two constructs due to the relatively unstable nature of the mini G_s_ I208 complex with the two receptors. Despite the relatively low coupling affinity of the I208A mutant, our native MS results demonstrate that the presence of ICL3 enhances the coupling of β1AR to mini Gs I208. Comparing the GDP release rates for β_1_AR, with and without ICL3, coupled to mini G_s_ I208 the results are closely similar allowing us to conclude that although coupling is less efficient the mutation does not impact GDP release rates (Supplementary Figure 9C).

Considering further this GDP release assay, wherein no difference between β_1_AR and β_1_AR_ICL3 was observed for wild-type or I208 mini G_s_, and our time-resolved HDX-MS data in which the first two coupling incubation times showed no effect of ICL3, we surmise that rather than influencing coupling at the early intermediate complex stage, ICL3 exerts its major effect later on the nucleotide-free state of the complex. To explore the mechanism further we therefore conducted a cell-based cAMP production assay to investigate the impact of ICL3 on β_1_AR signaling through the G_s_ pathway. To do this we used the Glosensor assay to compare HEK293 cells with overexpression of β_1_AR_ICL3 and the truncated construct. β_1_AR_ICL3 showed significantly stronger luminescence intensity (*p* < 0.0001) compared with truncated β_1_AR. This assay allows the inference that ICL3 plays an essential role in the canonical signaling of β_1_AR downstream of the early coupling events.

**Figure 6:**
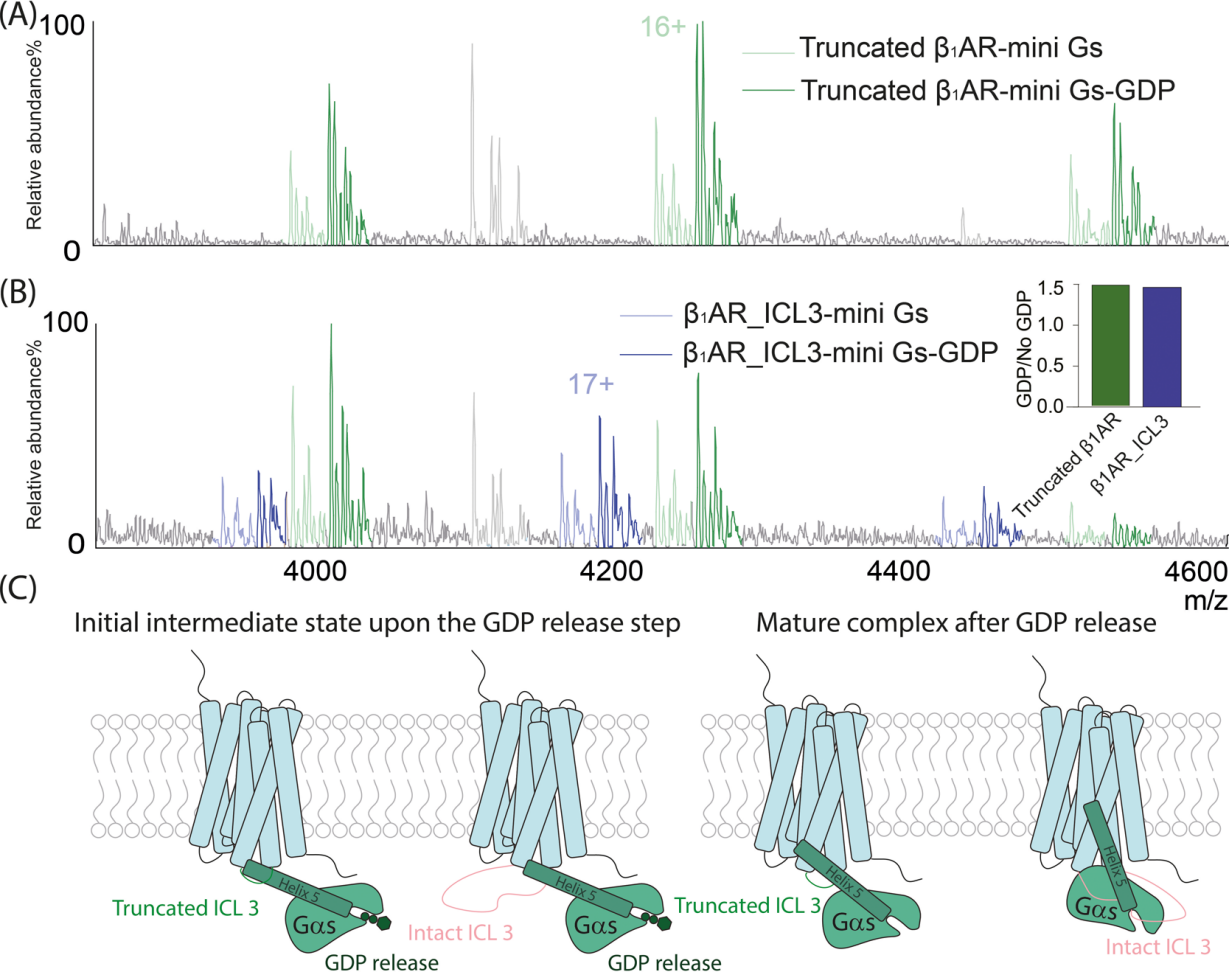
(A–B) Native mass spectra of the β_1_AR-mini G_s_-GDP complex. (A) The ratio of truncated β_1_AR:mini G_s_ was 1:1 (As we discussed above the spectrum was recorded immediately after addition of agonist < 10 s) (B) The ratio of truncated β_1_AR: β_1_AR_ICL3:mini G_s_ was 1:1:2. The peaks are assigned to truncated β_1_AR-mini G_s_ complex (pale green), the GDP-bound truncated β_1_AR- mini G_s_ complex (dark green), the β_1_AR_ICL3 mini G_s_ complex (light blue), and the GDP-bound β_1_AR_ICL3-mini G_s_ complex (dark blue). Peak intensity is quantified (inset bar graph) and shows that ICL3 does not change the binding affinity of the receptor to mini G_s_ GDP. (C) Illustrative figure showing that ICL3 does not influence the initial engagement of helix 5 of G_s_ to β_1_AR, at the intermediate state, prior to GDP release. Instead, ICL3 increases the affinity of β_1_AR to G_s_ by inducing different coupling poses of helix 5 of the G protein at a later stage, after GDP release.

To capture early coupling of a GDP-bound state via native MS we woud need to exlore complex formation in <60 s (Figure 5D). To do this, we first incubated β_1_AR and mini G_s_ and, after buffer exchange, added isoprenaline so that the solution could be analyzed immediately with a minimal incubation time. Under these conditions, the GDP-bound complex could be observed in native mass spectra (Figure 6A) allowing us to test whether β_1_AR_ICL3 has an effect on the affinity of this intermediate state. To compare the affinity of the two β_1_AR complexes to GDP, under the exact same MS and solution conditions, truncated β_1_AR, β_1_AR_ICL3 and mini G_s_ were incubated at a 1:1:2 ratio with excess isoprenaline. Native mass spectra were recorded, and the extent of mini G_s_-GDP versus apo mini G_s_ binding were assessed. The spectra are complicated with multiple split peaks due to nucleotide and metal ion binding. However, we are able to measure the ratio of GDP-bound (β_1_AR-mini G_s_ or β_1_AR_ICL3 mini G_s)_ to GDP-free (β_1_AR-mini G_s_ or β_1_AR_ICL3 mini G_s_) (Figure 6B). Comparable intensity ratios were observed (Figure 6B inset). Overall, this native MS result further supports our conclusions made from time-resolved HDX-MS and the GDP release assays that together show that ICL3 does not have a significant impact on the affinity of the complex during the initial stages of the coupling process, prior to GDP release. Rather the effect of ICL3 is realized on cAMP accumulation during the later stages of coupling.

## Conclusions

Herein, we investigated the intramolecular regulation of β_1_AR activity by ICL3 using native-MS, HDX-MS, a cell-based cAMP accumulation assay, and a GDP release assay. Native MS analysis showed that mini G_s_ preferentially couples to β_1_AR with an intact ICL3 loop (β_1_AR_ICL3) compared to β_1_AR without ICL3. Previous structural studies report extensive interactions between helix 5 of a G protein and intracellular regions of TM3, TM5, and TM6 of a GPCR.^38−42^ With HDX-MS analysis, we found that helix 5 of G_s_ can be used to probe the activity of GPCRs by qualitatively revealing the efficacy of various drugs to activate β_1_AR. We consider helix 5 of G_s_ to be a reliable reporter of the extent of activation since it forms part of the binding interface with the receptor.

It is interesting to compare our data with a previous NMR study that showed how chemical shift changes of M223^5.54^ and M296^6.41^ on β_1_AR correlated with ligand efficacy. The movement of these two residues was assigned to opening of the cytoplasmic end of β_1_AR for G protein coupling with the degree of conformational change likely regulated by drugs with different efficacy.^36^ NMR analyses of other class-A receptors, including β_2_AR and A_2A_R, led to the proposal that a distinct intermediate may exist between the inactive and active conformations.^21,40−42^ Nevertheless, detailed mechanisms of partial agonism have not been confirmed.^1^ Our study further suggests that the binding pose of helix 5 of mini G_s_, on the intracellular side of partial agonist bound β_1_AR, is different from the fully activated β_1_AR-mini G_s_ complex (Supplementary Figure 5C). Strikingly, under treatment of a full agonist, β_1_AR_ICL3 induces further protection of helix 5, upon complex formation, beyond that of agonist-bound β_1_AR. We propose therefore that ICL3 can induce a distinct binding orientation of helix 5 upon complex formation, that is not stabilized in the truncated construct. MD simulations give insights into the structural dynamics of complex formation between β_1_AR/β_1_AR_ICL3 and mini G_s_ and are consistent with our propsoal of two binding orientations of helix 5 of mini G_s_. In one of the binding poses (with ICL3) helix 5 shows a more compact interaction with the receptor. We propose that the presence of ICL3 of β_1_AR shifts the proportion of helix 5 to a relatively closed conformation which is stabilized by a hydrogen bond located within a highly protected patch (219–226) for enhanced coupling and alignment of the unstructured loop along the membrane.

Previous structural studies reported the existence of a transient intermediate complex in which GDP remains bound to the complex.^19−22^ A very recent study using time-resolved cryo-EM shows in intricate detail conformational changes of a GPCR-G protein complex after the addition of GTP.^46^ By contrast, our time-resolved HDX, with distinct β_1_AR-mini G_s_ incubation times, explores the role of ICL3 in the initial stages of G protein engagement, before the addition of GTP. Interestingly, rather than the higher protection of the helix 5 motif, as observed for long incubation times, no significant difference in deuterium exchange of helix 5 in complex with β_1_AR or β114_ICL3 is observed in the first 60 s of incubation. This observation was further supported by our GDP release assay, revealing that the rate of GDP release is not influenced by truncation of ICL3. Combined with our cell based signaling assay, ICL3 was proven to promote the upregulation of cAMP by agonist bound β_1_AR. Together these results imply that the regulatory effect of ICL3 occurs postnucleotide release on downstream signaling events.

In conclusion, we have shown that activation of β_1_AR via agonists leads to a level of engagement with mini G_s_ that is enhanced by the presence of ICL3. We propose that this enhanced engagement is achieved by changing the binding pose of mini G_s_ helix 5 to produce a proected patch on a more compact structure following GDP release (Figure 6C). Overall, therefore these results provide new insights into the function of this unstructured loop, post GDP release, in stabilizing coupling then promoting the cAMP downstream signaling. Collectively they shed new light on the intramolecular regulation of GPCR activation, with important consequences for drug design.

## Methods

Extended experimental and method details can be found in the Supporting Information (PDF). **For protein expression and purification**, β_1_AR, β_1_AR_ICL3 were overexpressed in Sf9 insect cells utilizing recombinant baculoviruses prepared using the dual expression vector pFastBac (Thermo Fisher). The engineered minimal G proteins, mini G_s_ construct R414 and mini G_s/i_ construct R43 were cloned into the pET15b plasmid for overexpression in *E. coli*. These proteins were purified by histogram affinity chromatography. **For native MS analysis**, β114, β114_ICL3, and mini G_s_ were buffer exchanged into MS Buffer (two times the CMC of Fos-Choline and 200 mM ammonium acetate) and analyzed on a Q-Exactive UHMR Hybrid Quadrupole-Orbitrap mass spectrometer (Thermo Fisher Scientific). Raw native MS spectra were deconvoluted and quantified by using UniDec software. **HDX-MS experiments** were performed on equipment from Waters Corporation, Manchester, UK. Purified receptors and mini G_s_ were diluted to concentrations of interest (the HDX process being sensitive to protein concentration^47^) using the equilibration buffer with different compositions. The **cAMP production assay** and **Time-resolved GDP assay** was performed by FLUOstar Omega Microplate Reader (BMG). **MD Simulations** were done using the GROMACS package with version 2022.4.^48^ Trajectory analysis was carried out using the gromacs tool and VMD.^49^

## Supporting Information Available

The Supporting Information contains the supplementary Figures S1–S9 and details of the experimental procedures. (PDF)
