# Supplementary material for: Coupling and Activation of the β1 Adrenergic Receptor - The Role of the Third Intracellular Loop
Source: J Am Chem Soc. 2024 Oct 3;146(41):28527–37. doi: 10.1021/jacs.4c11250 (PMC11487556; doi:10.1021/jacs.4c11250)
Supplement: Supplementary file 1 — ja4c11250_si_001.pdf [file ja4c11250_si_001.pdf]

**Supporting information**

# Coupling and activation of the β1 adrenergic receptor - the role of the third intracellular loop

Xingyu Qiu[1,2], Kin Chao[3], Siyuan Song[1,2], Yi-Quan Wang[4], Yi-An Chen[4], Sarah L. Rouse[3], Hsin Yung Yen[1,4*], Carol V. Robinson[1,2*]

AUTHOR ADDRESS

[1] Physical and Theoretical Chemistry Laboratory, Department of Chemistry, University of Oxford, Oxford, OX1 3QZ, UK

[2] Kavli Institute for Nanoscience Discovery, Dorothy Crowfoot Hodgkin Building, University of Oxford, Oxford, OX1 3QU, UK

[3] Department of Life Sciences, Imperial College London, South Kensington Campus, London, SW7 2AZ, UK

[4] Institute of Biological Chemistry, Academia Sinica, Taipei, 115024, Taiwan

## Supplementary Figures

Supplementary Figure 1

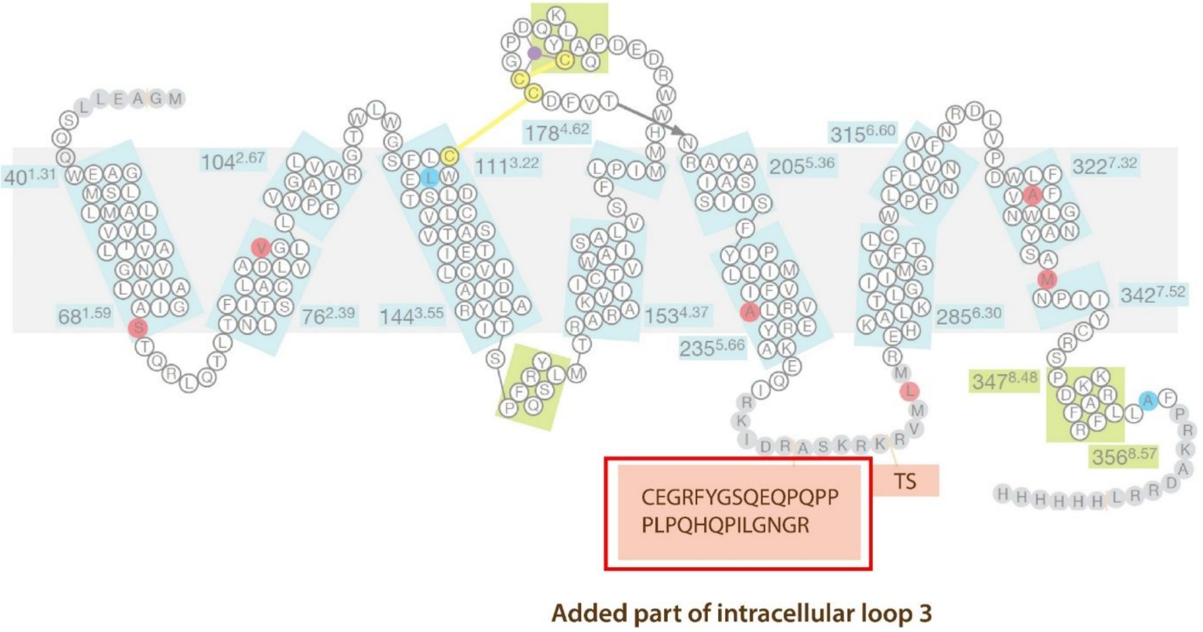


**Supplementary Figure 1 (A) snake plot of the $\beta_1$AR sequence including the sequence of ICL3**. The sequence of ICL3 used in our study is highlighted and added to β114-E130W (known here as truncated β114). Figure adapted from Warne *et al.* [18]

Supplementary Figure 2

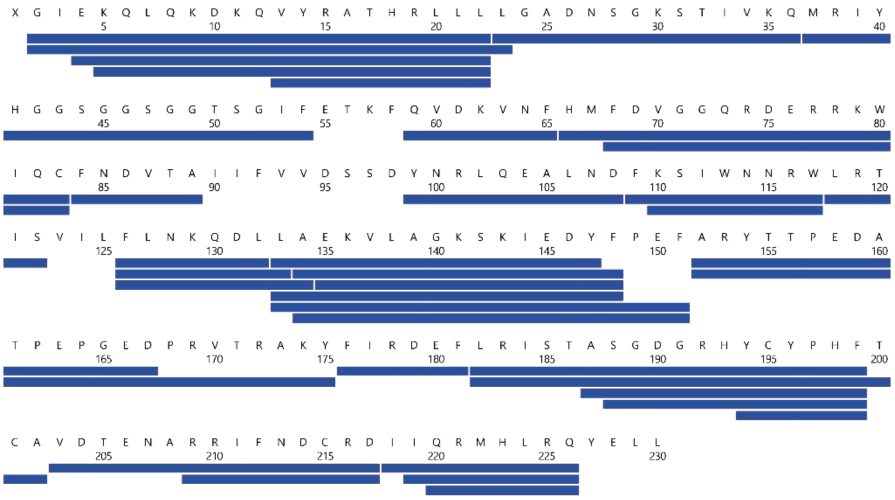


**Supplementary Figure 2. Sequence coverage of mini $G_s$ following pepsin digestion and peptide identification (see SI Appendix).** 92% sequence coverage of mini $G_s$ is obtained.

Supplementary Figure 3

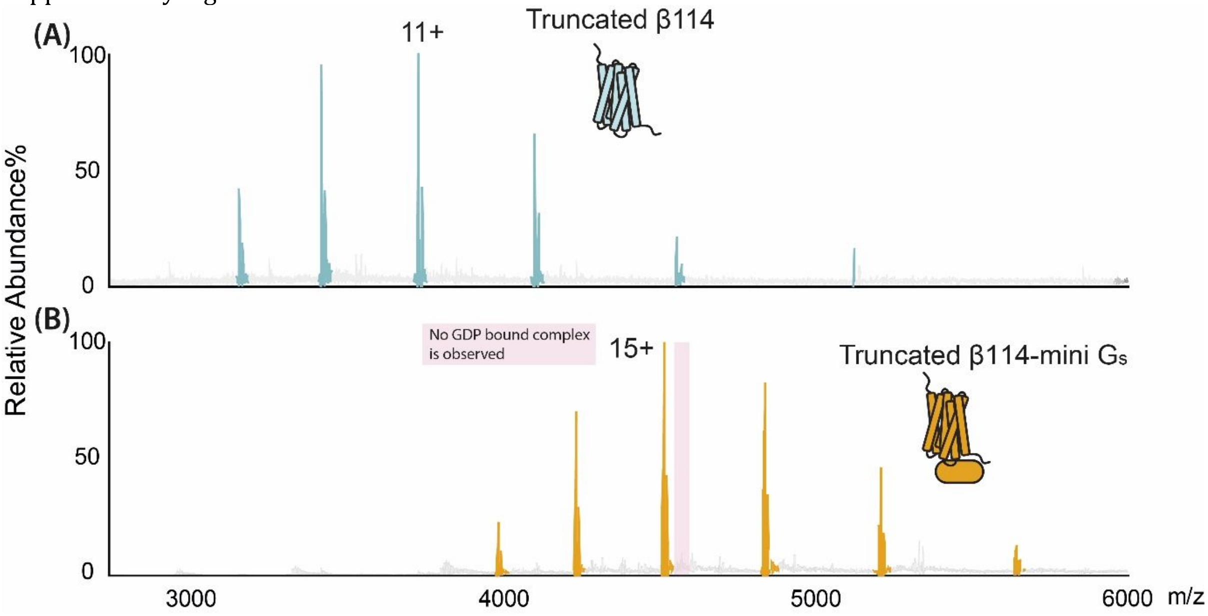


**Supplementary Figure 3. Native mass spectra 20 min after incubation of truncated $\beta_1$AR alone (A) and in complex with mini $G_s$ ($\beta_1$AR (1.5 μM): mini $G_s$ (1.5 μM) ratio is 1:1.2) (B).** 100% coupling is observed under these conditions with no free receptor detected in the presence of mini $G_{s.}$ Peaks assigned to free mini Gs are below the 3000 m/z cutoff. No GDP bound complex was observed. Only the mature complex is detected after 20 mins incubation.

Supplementary Figure 4

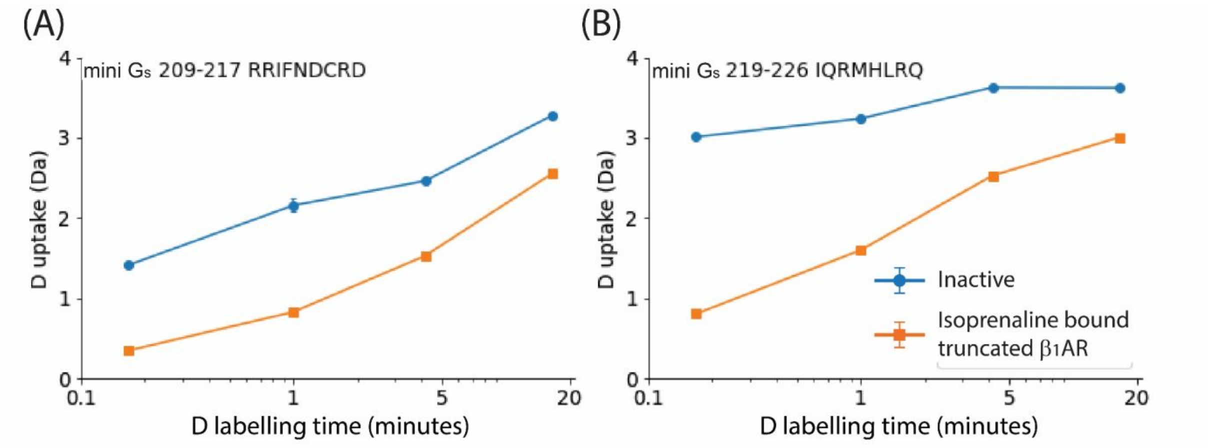


**Supplementary Figure 4. Deuterium uptake of two $G_s$ peptides from the complex formed between mini $G_s$:$\beta_1$AR without ICL3.** Results are plotted as a function of deuterium labelling time from 10 s to 1000 s for two selected peptides covering **A.** the N terminal residues and **B.** the C terminal residues of mini $G_s$ helix 5. Error bars are generated according to the standard deviation calculated from three independent repeats. A student's *t*-test was used to evaluate the statistically significant differences ($p<0.01$).

Supplementary Figure 5

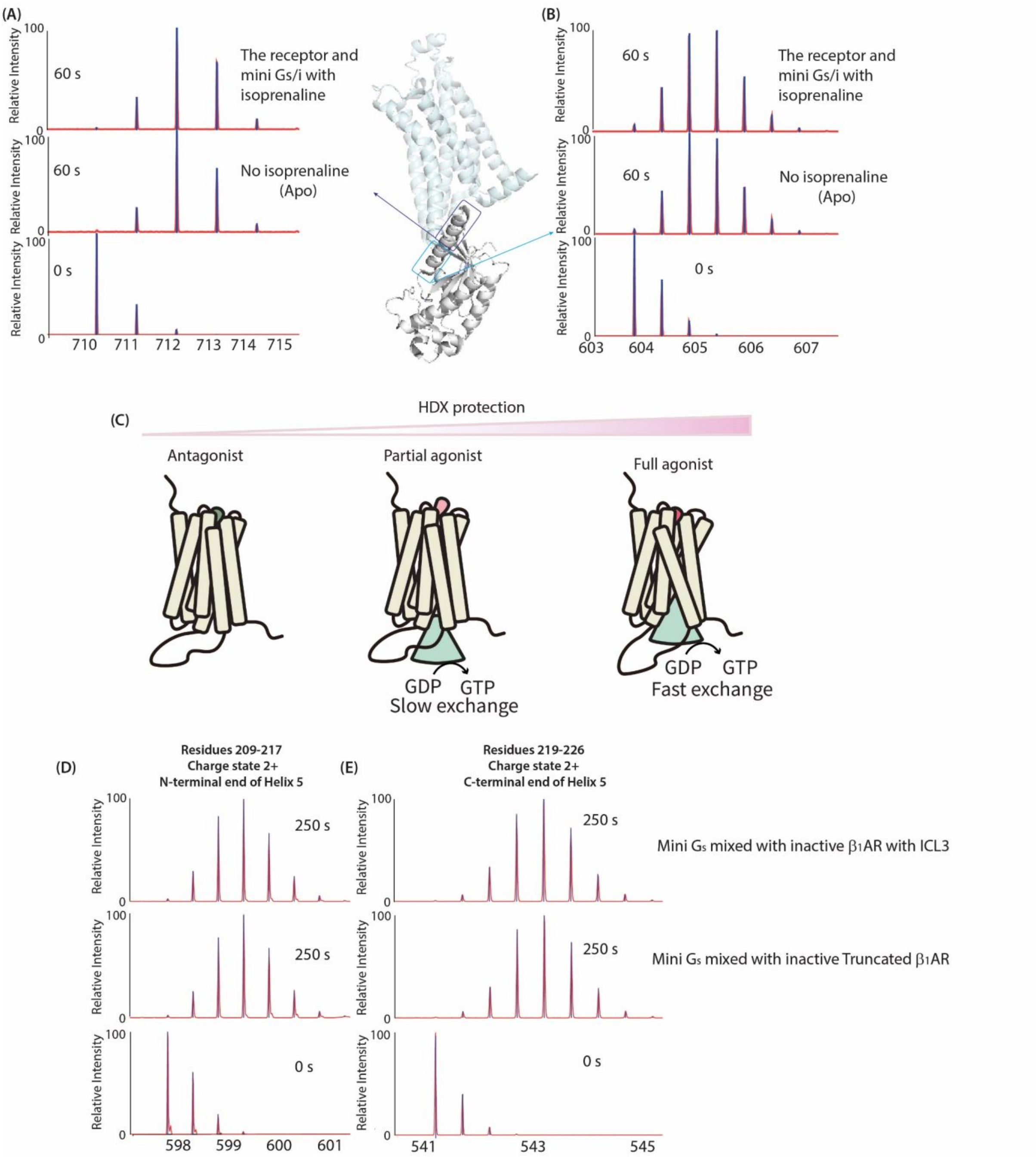


**Supplementary Figure 5 (A-B) Mass spectra showing deuterium uptake for two helix 5 peptides 'RDCGLF' and 'ARRIFNDVTD' in complex with mini $G_{s/i}$:$\beta_1$AR without ICL3 and control experiments for both constructs in the absence of agonists.** Deuterium uptake after 60 s labelling time of (**A**) the C-terminal and (**B**) the N-terminal peptides of helix 5 of mini $G_{s/i.}$ Helix 5 does not change after incubation with isoprenaline bound $\beta_1$AR. **(C)** Illustrative figure shows partially active and active states of a GPCR regulated by the efficacy of drugs, adapted from a previous NMR study[1]. **(D-E)** Control experiments in the absence of isoprenaline confirm that the two helix 5 peptides exhibit no protection against hydrogen exchange when compared with the isoprenaline bound states (Figure 2A and 2B truncated construct) and (Figure 3A and 3B $\beta_1$AR-ICL3) (main manuscript).

Supplementary Figure 6

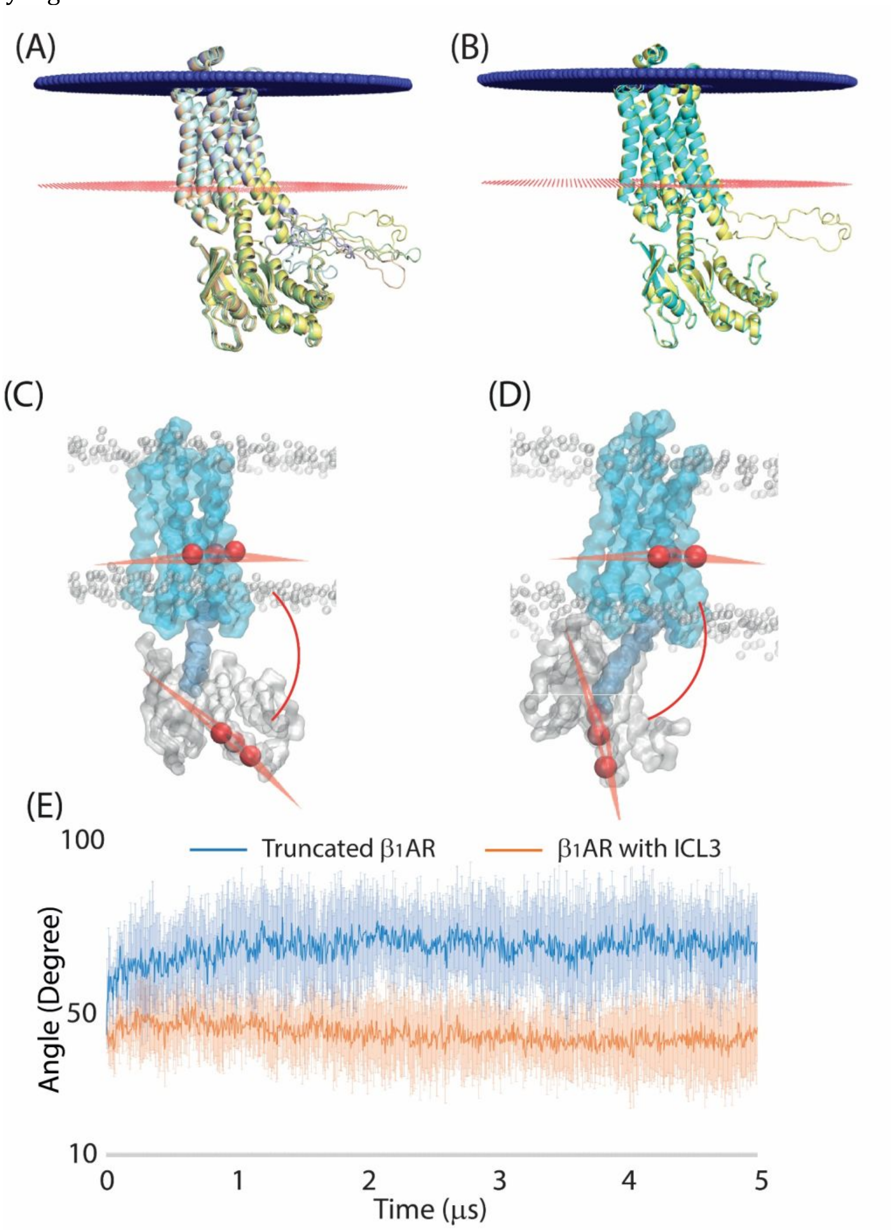


**Supplementary Figure 6 Initial models used for MD based on the *M. gallopavo* $\beta_1$AR cryo EM structure of dobutamine–$\beta_1$AR–Gs (8DCR))**[2] **(A)** 5 ICL3 models aligned, showing the five initial ICL3 loop conformation generated from MoMA[3] **(B)** Aligned model showing apo $\beta_1$AR_ICL3 (yellow) and truncated $\beta_1$AR (cyan). **(C-D)** MD simulations show two binding orientations of mini $G_s$ relative to $\beta_1$AR. Relative mini $G_s$ orientation throughout the simulations is shown in **(C)** and **(D).** The angle formed between the plane by three residues in $\beta_1$AR (I222, G297, F338) and the plane formed by three residues in mini $G_s$ (S97, K129 and K136) were measured during the simulations. **(E)** The averaged values for the angle were calculated for $\beta_1$AR with ICL3 (orange) and the truncated $\beta_1$AR (blue). Results show that mini $G_s$ coupled to $\beta_1$AR _ICL3 adopts a lower angle in general (the orientation shown in **(C)**). The standard deviation is shown (transparent lines) and the full angle measurement for each individual cgMD runs can be found in **Supplementary Figure 7**.

Supplementary Figure 7

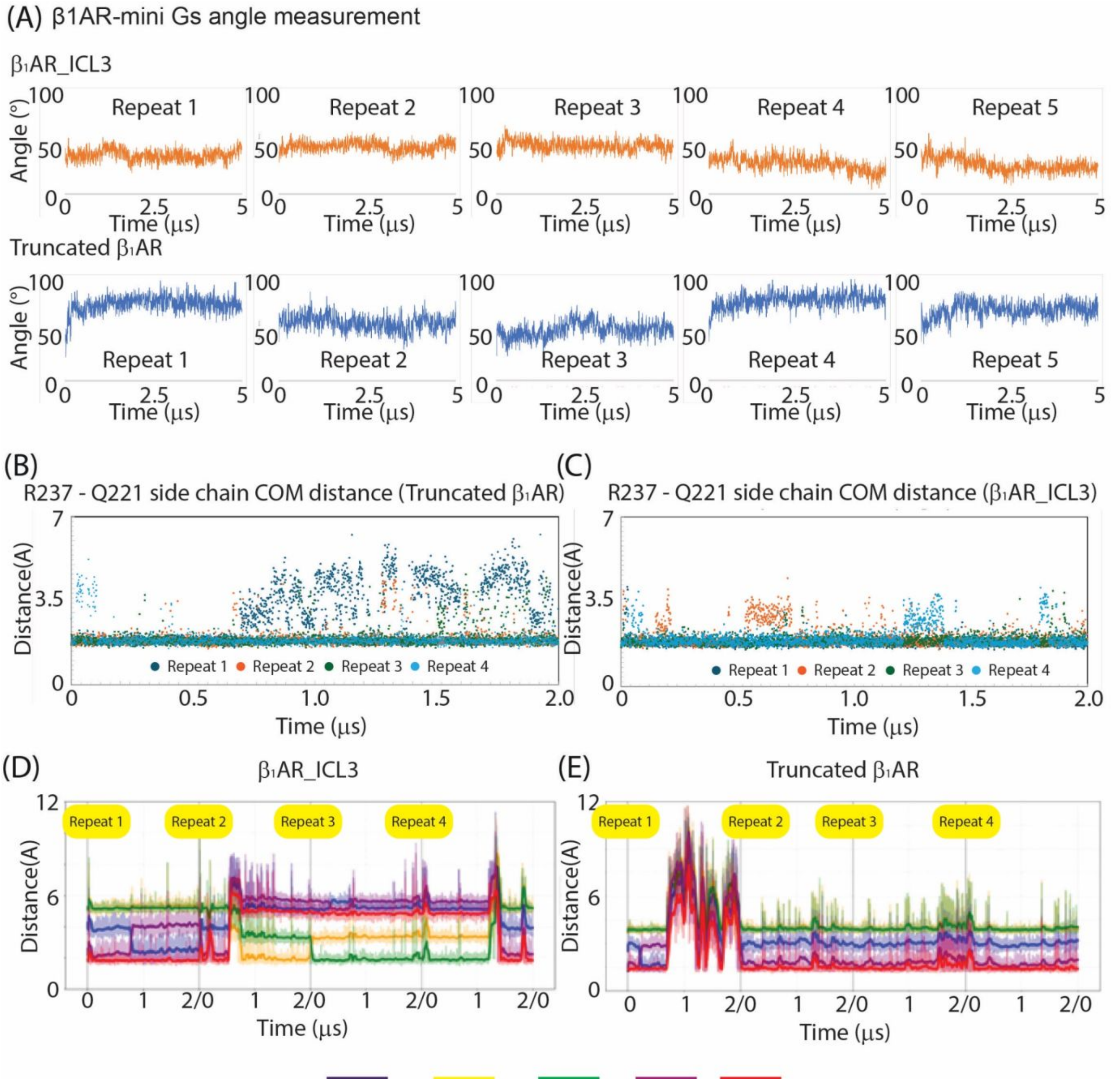


**Supplementary Figure 7 (A) $\beta_1$AR-mini Gs angle measurement in cgMD** The angle formed between by three residues in the plane of $\beta_1$AR (I222, G297, F338) and the plane formed by three residues in mini $G_s$ (S97, K129 and K136) is measured as a function of simulation time. The following color scheme was used throughout $\beta_1$AR_ICL3 (orange) and truncated $\beta_1$AR (blue). **(B-E) Q237 ($\beta_1$AR) and R221 (mini Gs) distance measurement in atMD (B-C)** The distance between the center of mass (COM) of the side chains of Q237 (β1AR) and R221 (mini Gs) for β1AR_ICL3 **(B)** and Truncated_β1AR **(C)** throughout the simulation, with each repeat colored differently. **(D-E)** The distance between the hydrogen bond with the oxygen in Q237 (β1AR) and the five different hydrogens of R221 (mini $G_s$), with each distance colored differently based on the specific hydrogen. Rolling average is shown as a transparent line.

Supplementary Figure 8

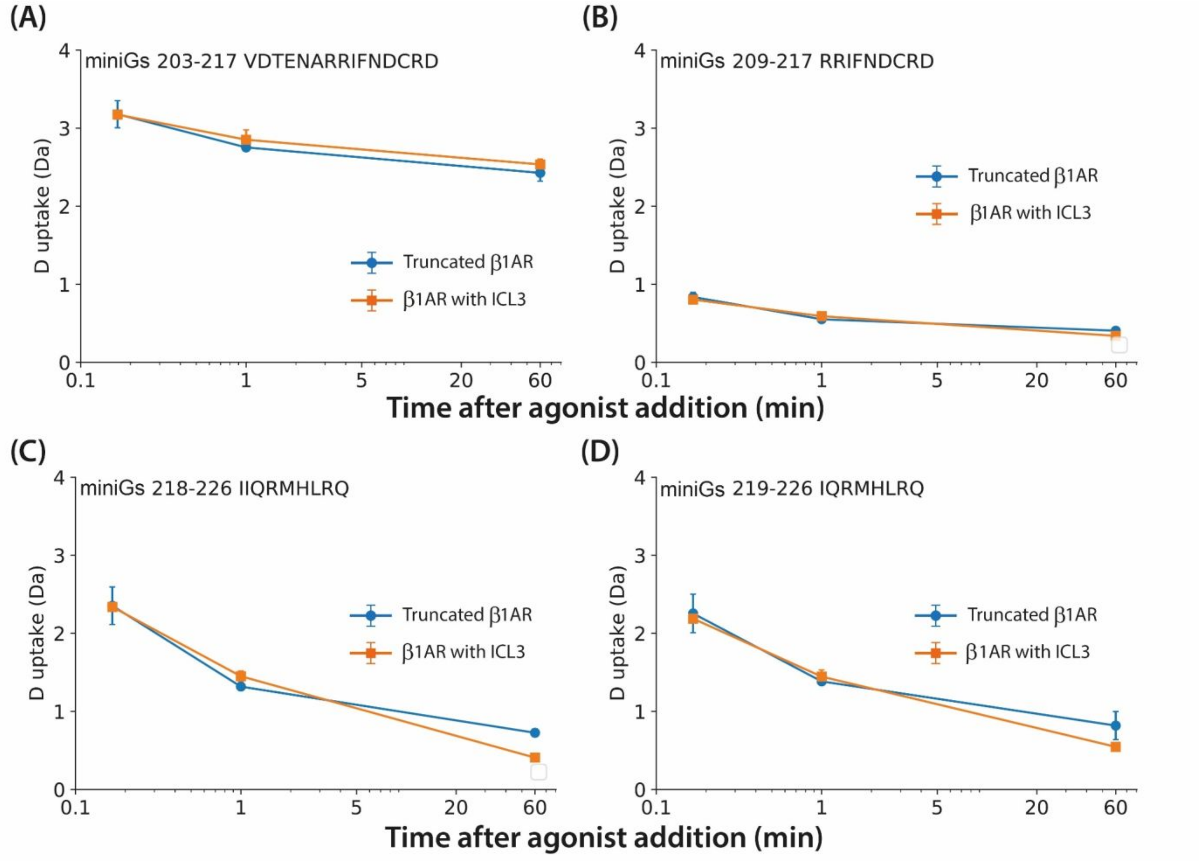


**Supplementary Figure 8 Comparison of time-resolved HDX-MS for truncated $\beta_1$AR : mini $G_s$ and $\beta_1$AR_ICL3:mini $G_s$.** Deuterium uptake for various helix 5 mini $G_s$ peptides are plotted as a function of the coupling incubation time (10 seconds, 1 minute and 60 minutes) for **(A-B)** the N terminal residues and **(C-D)** the C terminal residues. The deuterium labelling time is 10 s in each case. The error bars are generated according to the standard deviation calculated from three independent repeats.

Supplementary Figure 9

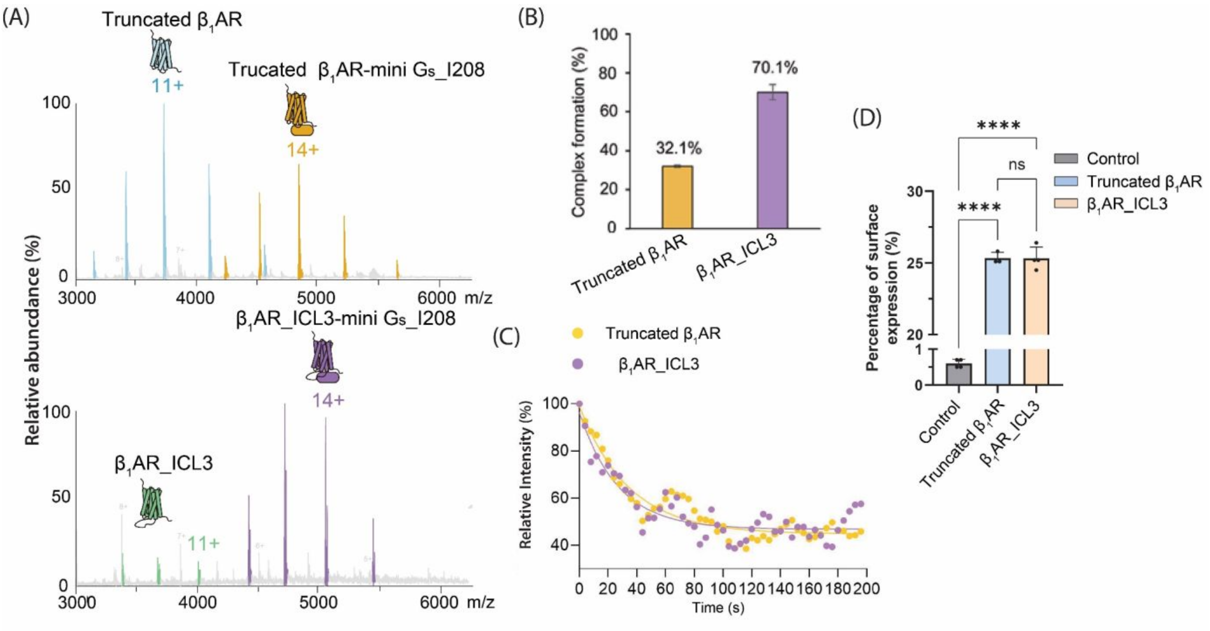


**Supplementary Figure 9 (A-B) ICL3 of $\beta_1$AR promotes the complex formation with mini $G_s$ I208. (A)** Native mass spectra for mini $G_s$ I208 coupling with truncated $\beta_1$AR (upper spectrum) and $\beta_1$AR_ICL3 (bottom spectrum). The truncated $\beta_1$AR and $\beta_1$AR_ICL3 were colored in blue and green, whereas their correspondent complexes with mini $G_s$ I208 were marked in yellow and purple, respectively. **(B)** Quantification of complex formation between mini $G_s$ I208 and $\beta_1$AR variants. The relative percentage of $\beta_1$AR – mini $G_s$ complex was plotted for truncated $\beta_1$AR (yellow) and $\beta_1$AR_ICL3 (purple). **(C) ICL3 does not influence the GDP release rate of mini $G_s$ I208 coupled to $\beta_1$AR.** The GDP release assay of mini $G_s$ I208 against truncated $\beta_1$AR (yellow) and $\beta_1$AR_ICL3 (purple) upon isoprenaline treatment. The relative fluorescent intensity is plotted by a function of time and the decrement curve is fitted with a one-phase decay equation to indicate the release of nucleotide. **(D) Cell surface expression analysis of β1AR_ICL3, Truncated β1AR, and control determined by flow cytometry.** Error bars are generated according to the standard deviation calculated from four independent biological repeats (control), four independent biological repeats ($\beta_1$AR_ICL3), three independent biological repeats (Truncated $\beta_1$AR). A one-way ANOVA test was used to evaluate the statistically significant differences. Truncated $\beta_1$AR and $\beta_1$AR_ICL3 showed similar levels of cell surface expression after transfection.

## Methods

### Expression and purification of $\beta_1$AR, $\beta_1$AR_ICL3, and mini $G_s$ and mini $G_{s/i}$

### Truncated $\beta_1$AR (β114), $\beta_1$AR_ICL3 (β114_ICL3)

The *M. gallopavo* β114-E130W (β114) and β114-E130WIC3 (β114_ICL3) were overexpressed in Sf9 insect cells utilizing recombinant baculoviruses prepared using the dual expression vector pFastBac (Thermo Fisher). The insect cell pellet was resuspended in 20 mM Tris-HCL 1mM EDTA, a protease inhibitor cocktail (Roche). The homogenous mixture was passed twice through an M-110 PS microfluidizer (Microfluidics) at 8000 psi. The cell lysate was then centrifuged at 9000g for 20 min at 4 °C for the removal of insoluble cell residues. The supernatant was ultracentrifuged at 200,000g for 1 h at 4 °C. The membrane fraction was collected and resuspended in a washing buffer (20 mM Tris-HCL 1mM EDTA) followed by ultracentrifugation for 1 h. The membrane fraction was then further resuspended in 20Mm Tris-HCL, 0.2mM EDTA followed by 1 h ultracentrifugation. After removing the supernatant, the protein was solubilized from the membrane fraction with 20 mM Tris-HCl pH8, 350 mM NaCl, 3 mM imidazole, 1.5% (w/v) n-dodecyl-β-D-maltopyranoside (DDM, Anatrace). The supernatant containing the membrane protein was clarified by centrifugation at 32,000 rpm for 1 h. The supernatant was then filtered before loading onto a 5 ml HisTrap-TALON crude column (GE Healthcare, Piscataway, NJ) equilibrated in 20 mM Tris–HCl pH 8, 350 mM NaCl, 3 mM imidazole and 0.05% DDM. The column was washed with ten column volumes of equilibration buffer. The bound receptor was eluted by a gradient of 20 mM Tris–HCl pH 8, 350 mM NaCl, 250 mM imidazole and 0.05 % DDM in three column volumes. The fractions containing receptors were collected and concentrated to the final concentration of 2-3 mg/ml followed by buffer exchange to the equilibration buffer (20 mM Tris–HCl pH 8, 350 mM NaCl, 3 mM imidazole and 0.05% DDM, 5% glycerol).

### Mini $G_s$ and mini $G_{s/i}$

The engineered minimal G proteins, mini $G_s$ construct R414 and mini $G_{s/i}$ construct R43 were cloned into the pET15b plasmid for overexpression in *E. coli*. The constructs are designed with an N-terminal histidine tag and a TEV protease cleavage site to promote purification and tag removal. The plasmid was transformed in *E. coli* strain BL21-Gold (New England Biolabs). Colonies were inoculated into 150 ml LB media (supplemented with 0.2% glucose, 30 µg/ml kanamycin) and grown overnight at 16-20 h at 37 °C, shaking at 700 rpm. 500 mL of TB (supplemented with 0.2% glucose, 30 µg/ml kanamycin, and 5mM $MgSO_4$) in 2-litre shaker flasks was inoculated with 15 ml of cells and grown at 37 °C until the $OD_{600nm}$ reached 0.6-0.8. Expression of mini G was induced by the addition of IPTG to give a final concentration of 50 µM. The mixture was then incubated for 16-20h at 25 °C, shaking at 200 rpm. The cell pellet was harvested by centrifugation at 5,000xg for 10 min at 4 °C. Cell pellets were resuspended in 40 mM HEPES, pH 7.5, 100 mM NaCl, 10mM imidazole, 10% v/v glycerol, 5 mM $MgCl_2$, 50 mM GDP, protease inhibitor cocktail (Roche), DNase I (50 µg/ml), lysozyme (50 µg/ml), and TCEP (100 µM). The homogenous mixture was passed 3 times through an M-110 PS microfluidizer (Microfluidics) at 15000 psi. The cell lysate was centrifuged at 38000g for 45 minutes at 4 °C for the removal of insoluble cell residues. The supernatant was filtered before loading onto a 5 ml HisTrap-TALON crude column (GE Healthcare, Piscataway, NJ) equilibrated in 20 mM HEPES, pH 7.5, 500 mM NaCl, 40mM imidazole, 10% v/v glycerol, 1 mM $MgCl_2$, 50 µM GDP. The column was washed with ten column volumes of equilibration buffer. The bound protein was eluted by a gradient of 20 mM HEPES, pH 7.5, 100 mM NaCl, 500 mM imidazole, 10% v/v glycerol, 1 mM $MgCl_2$, 50 µM GDP in three column volumes. The fractions containing proteins were collected and concentrated to the final concentration 1 mg/ml followed by buffer exchange to the equilibration buffer. TEV protease was added to give a TEV: mini $G_s$ ratio of 1:20 w/w for the cleavage of the histidine tag using TEV protease. TCEP (1mM) was also added. The cleaved tag, protease and undigested mini G proteins were removed by reverse IMAC purification on $Ni^{2+}$-NTA. Proteins were concentrated to 2 mg/ml in 20 mM HEPES, pH 7.5, 100 mM NaCl, 10% v/v glycerol, 1 mM $MgCl_2$, and 10 mM GDP.

## Native MS analysis

### Mini $G_s$ coupling to the truncated $\beta_1$AR and $\beta_1$AR_ICL3

Mini $G_s$ was incubated with purified **truncated $\beta_1$AR** and **$\beta_1$AR_ICL3** at a 1:1:1 molar ratio in the coupling buffer (10 mM HEPES, 10 mM Tris-HCl, pH7.4, 200 mM NaCl, 1mM $MgCl_2$, 5 mM GDP and 0.05 % DDM) containing 200 μM isoprenaline. The sample was then buffer exchanged into MS Buffer (two times the CMC of Fos-Choline and 200 mM ammonium acetate) using a centrifugal buffer exchange device (Micro Bio-Spin 6, Bio-Rad) as previously described (Laganowsky *et al.*)[4]. After detergent exchange and desalting, the protein solutions (2-3 μL) were loaded into an in-house prepared gold-coated Clark borosilicate capillary (Harvard Apparatus). The sample was analysed using a Q-Exactive UHMR Hybrid Quadrupole-Orbitrap mass spectrometer (Thermo Fisher Scientific). The instrument has been modified and optimised for detecting membrane proteins. The instrument parameters for MS were: 1.4 kV capillary voltage, S-lens RF 100%, quadrupole selection from 2000 to 8000 m/z range, collisional activation in the HCD cell 200 V source fragmentation 0 V, in-source trapping 0 V for analysis in positive mode. The ion transfer optics in positive mode was set as follows: injection flat-pole 5 V, inter-flat-pole lens 4 V, bent flat-pole 2 V, transfer multipole 0 V; opposite polarities were used for negative mode acquisition. The resolution of the instrument was 17,500 at m/z=200 (transient time of 64 ms), argon pressure in the HCD cell was maintained at approximately $8 \times 10^{-10}$ mbar and source temperature was maintained at 100 °C. The noise level was set at 3 rather than the default value of 4.64. Calibration of the instrument was performed using a 10 mg/ml solution of caesium iodide in water. The population of monomers and complexes was quantified by UniDec software based on the peak intensity.

To investigate the immediate coupling event at the initial stage, purified truncated $\beta_1$AR, $\beta_1$AR_ICL3, and mini $G_s$ were buffer exchanged separately into 200 mM ammonium acetate buffer pH 8 containing the mixed micelle preparation and 5 mM GDP. The receptors and mini $G_s$ are mixed at the molar ratios mentioned in section the main manuscript and the protein mixture was introduced into Mass Spectrometry immediately after adding isoprenaline to a final concentration of 200 μM. Spectra were acquired for 1 min and the relative percentage of $\beta_1$AR-mini $G_s$ with and without GDP was quantified by comparing the peak intensities.

## HDX-MS

### General procedures

The HDX-MS experiments were performed on equipment from Waters Corporation, Manchester, UK. The purified protein was diluted to a concentration of interest using the protein buffer (equilibration buffer) with different compositions.

Five μL of the protein solution was incubated with 55 μL of the deuterated buffer for the time course of interest at 20 °C, then quenched by 60 μL of quench buffer at pH of 2.0 and 4 °C. The solution (80 μL) was then injected into the Waters nano ACQUITY UPLC System and digested online by passing through the Waters Enzymate™ BEH Pepsin Column at 20 °C. Peptides were trapped and desalted on a Waters BEH C18 VanGuard pre-column with buffer A (0.1 % formic acid pH 2.8) for 3 min at a flow rate of 100 μL/min, then separated by BEH C-18 analytical column with a linear gradient of buffer B (5–80% gradient of acetonitrile with 0.1 % formic acid) at a flow rate of 40 μL $min^{-1}$. The temperature was reduced to 4 °C after digestion to minimise back exchange. Waters Synapt G2Si HDMS was used to acquire the MS data of yielded peptides.

To acquire the MS data of yielded peptides, Waters Synapt G2Si HDMS is coupled to Waters nano ACQUITY UPLC System. The electrospray ionization source was operated in the positive ion mode. $MS^E$ data were acquired with a 20-30 V trap collision energy ramp for the high-energy acquisition of product ions. The system is washed with clean buffer (1.5 M Gu-HCl, 4 % MeCN, 0.8% formic acid) between each analysis.

Sequence coverage and HDX data were analysed by ProteinLynx Global Server 2.5.1 (PLGS Waters Corp. Manchester, UK) and DynamX 3.0. Peptide identification was achieved from $MS^E$ data of the non-deuterated proteins using ProteinLynx Global Server 2.5.1 (PLGS Waters Corp. Manchester, UK. The resulting peptides were selected using DynamX 3.0 with the selection criteria: minimum intensity of

1000, minimum and maximum peptide sequence length of 5 and 30, respectively, minimum products per amino acid of 0.2, a minimum score of 5, and a maximum $MH^+$ error threshold of 15 p.p.m.)

**HDX-MS of mini $G_s$ coupling to $\beta_1$AR**

Prior to HDX labelling, the purified truncated $\beta_1$AR or $\beta_1$AR_ICL3 were incubated with mini $G_s$ at a 1:1.2 molar ratio at 20 °C in the coupling buffer (10 mM HEPES, 10 mM Tris-HCl, pH7.4, 200 mM NaCl, 1mM $MgCl_2$, 5 mM GDP and 0.05% DDM) with 200 μM isoprenaline for at least 20 minutes. 5 μL of the solution (20 μM of mini $G_s$, 24 μM of $\beta_1$AR with or without ICL3, with 200 μM of isoprenaline) was labelled with 55 μL of the deuterated buffer (20 mM Tris–HCl pD 8, 350 mM NaCl, 3 mM imidazole and 0.05% DDM) for a time course of interest, then quenched by 60 μL of quench buffer (20 mM Tris–HCl pH 2, 15mM TCEP) and 4 °C. The following procedures of HDX-MS analysis are as stated in the general procedures.

To conduct the concentration-dependent HDX-MS, the purified truncated $\beta_1$AR or $\beta_1$AR_ICL3 was incubated with mini $G_s$ at the molar ratio of interest at 20 °C in the coupling buffer (10 mM HEPES, 10 mM Tris-HCl, pH7.4, 200 mM NaCl, 1mM $MgCl_2$, 5 mM GDP and 0.05% DDM) with 200 μM isoprenaline for at least 20 min. The concentration of mini $G_s$ was fixed at 20 μM while concentrations of receptors varied from 0 to 48 μM. 5 μL of the solution was then incubated with 55 μL of the deuterated buffer (20 mM Tris–HCl pD 8, 350 mM NaCl, 3 mM imidazole and 0.05% DDM) for 250 s, then quenched by 60 μL of quench buffer (20 mM Tris–HCl pH 2, 15Mm TCEP) and 4 °C. HDX-MS analysis was carried out as stated in the general procedures.

To conduct the time-resolved HDX-MS**,** purified truncated $\beta_1$AR or $\beta_1$AR_ICL3 was incubated with mini $G_s$ at a 1:1.2 molar ratio at 20 °C in the coupling buffer (10 mM HEPES, 10 mM Tris-HCl, pH7.4, 200 mM NaCl, 1mM $MgCl_2$, 5 mM GDP and 0.05% DDM) with 200 μM isoprenaline for an incubation time that varied from 10 s to 1 hr. 5 μL of the mixture were labelled with 55 μL of the deuterated buffer (20 mM Tris–HCl pD 8, 350 mM NaCl, 3 mM imidazole and 0.05% DDM) for 10 s or 60 s, then quenched by 60 μL of quench buffer (20 mM Tris–HCl pH 2, 15 mM TCEP) and 4 °C. HDX-MS analysis was carried out as stated in the general procedures. The deuteration level for each peptide was analyzed and quantified by DynamX 3.0. HDX versus labelling time graphs were plotted using MATLAB.

**Time-resolved GDP assay**

Truncated $\beta_1$AR or $\beta_1$AR_ICL3 (5 μL, 48 μM) was mixed with mini $G_s$ (5 μL, 40 μM) and Bodipy-GDP (10 μL, 500 nM) in 10 mM HEPES, 10 mM Tris-HCl, pH7.4, 200 mM NaCl, 1mM $MgCl_2$, and 0.05% DDM. The mixture was loaded into a 384-well plate. The measurement started immediately after the addition of isoprenaline in water (1 μL, 1 mM). The fluorescence intensity was determined at 20 °C by FLUOstar Omega Microplate Reader (BMG). The excitation and emission filter were set at 485 nm and 520 nm respectively. The total measurement time was set at 100 s with 1000 times of measurement (Interval time: 0.1s, Intervals number: 1000). Orbital shaking was applied to the samples. Every 30 successive FL intensities (A.U) recorded were averaged to yield recorded FL intensity in 1.5 second time periods.

**cAMP accumulation assay**

For truncated $\beta_1$AR and $\beta_1$AR_ICL3, HEK293T cells in six-well plate was co-transfected with a pcDNA3.1 plasmid expressing $\beta_1$AR and a luciferase-based cAMP biosensor, pGlosensor-22F (Promega) mixing at a ratio of 2:1. Concentrations of plasmids were adjusted optimised so that the surface expression levels of truncated $\beta_1$AR and $\beta_1$AR_ICL3 are comparable confirmed by flow cytometry exploiting an anti-HA antibody conjugated with Alexa Fluor® 647 (Supplementary Figure 9D). The assay was performed 24 hours after transfection, Expi293 expression medium plus 2% (v/v) Glosensor assay reagent (Promega) was added to well and incubated for 3 hours. Immediately after isoprenaline addition, luminescence was measured using FLUOstar Omega Microplate Reader (BMG).

**Molecular dynamics simulations**

Model building: The *M. gallopavo* $\beta_1$AR cryoEM structure 8CDR was used in this study[2]. The missing ICL3 loop was modelled using MoMA-LoopSampler[3] to generate 5 different initial ICL3 conformations. Truncated ICL3 models were generated using ColabFold webserver,[5] using AlphaFold2 with pdb100

template mode[6]. Mini $G_s$ was aligned to the $G_s$ in the cryoEM model (a full alignment of all the models used can be found in Supplementary Figure 8).

Coarse-grained molecular dynamics simulations (cgMD): $\beta_1$AR and mini $G_s$ were coarse-grained using Martinize2[7] with the Martini 2.2 forcefield[8]. The ElNeDyn elastic network restraint[9] with an elastic bond force constant of 500 kJ/mol/nm2 and an upper cut-off of 0.9 nm were applied separately to both $\beta_1$AR and mini $G_s$. The transmembrane region of the GPCR was predicted using PPM 3.0 Web Server[10] and embedded into an asymmetric lipid membrane bilayer in a 25 x 25 x 19 $nm^3$ box using insane.py.[11] Each system was solvated using MARTINI water[12] and 0.15 M NaCl followed by minimisation and equilibration steps. All the production simulations were 5 μs in length and 5 repeats were performed for both apo and truncated state. The v-rescale thermostat (tau 1.0 ps)[13] and the Parrinello–Rahman barostat (tau 12.0 ps)[14] were used to maintain temperature (303.15 K) and pressure (1 bar) in the production runs. Simulations were done using the GROMACS package with version 2022.4.[15] Trajectory analysis was done using gromacs tool and VMD.[16] Mammalian plasma membrane composition as used by Song et al[17] was as follows. Upper leaflet:
POPC(20%), DOPC(20%), POPE (5%), DOPE(5%), POSM(15%), GM3(10%), CHOL(25%). Lower leaflet: POPC(5%), DOPC(5%), POPE(20%), DOPE(20%), CHOL(25%), POPS(8%), DOPS(7%), PIP2(10%).

Atomistic molecular dynamics simulations (atMD): All input files were prepared using CHARMM-GUI membrane builder[18] with CHARMM36m[19] forcefield using the *M. gallopavo* β114-E130W (β114) and β114-E130WIC3 (β114_ICL3) sequences. The TIP3P[20] water model was used and each system was solvated with 0.15 M NaCl. The standard 6 steps equilibration and production settings implemented in CHARMM-GUI were used. The Nose-Hoover thermostat[21] was used to maintain temperature (303.15 K) and the Parrinello–Rahman barostat[22] with a compressibility of 4.5 x $10^{-5}$/ bar was used to maintain pressure (1 bar). The long-range electrostatics was calculated using Particle Mesh Ewald (PME)[23] method with a cutoff distance of 1.2 nm. The LINCS[24] algorithm was used to constraint hydrogen bonds. The same membrane composition used in cgMD was also used. atMD production rus were 2us in length, with 4 repeats for both truncated β1AR and β1AR_ICL3.

# REFERENCES


(1) Solt, A. S.; Bostock, M. J.; Shrestha, B.; Kumar, P.; Warne, T.; Tate, C. G.; Nietlispach, D. Insight into Partial Agonism by Observing Multiple Equilibria for Ligand-Bound and Gs-Mimetic Nanobody-Bound B1-Adrenergic Receptor. *Nat. Commun.* **2017**, *8* (1), 1795. DOI: 1038/s41467-017-02008-y.

(2) Su, M.; Paknejad, N.; Zhu, L.; Wang, J.; Do, H. N.; Miao, Y.; Liu, W.; Hite, R. K.; Huang, X. Y. Structures of B1-Adrenergic Receptor in Complex with Gs and Ligands of Different Efficacies. *Nat. Commun.* **2022**, *13* (1), 4095. DOI: 10.1038/s41467-022-31823-1.

(3) Barozet, A.; Molloy, K.; Vaisset, M.; Zanon, C.; Fauret, P.; Siméon, T.; Cortés, J. MoMA-LoopSampler: A Web Server to Exhaustively Sample Protein Loop Conformations. *Bioinformatics* **2022**, *38* (2), 552–553. DOI: 10.1093/bioinformatics/btab584.

(4) Laganowsky, A.; Reading, E.; Hopper, J. T. S.; Robinson, C. V. Mass Spectrometry of Intact Membrane Protein Complexes. *Nat. Protoc.* **2013**, *8* (4), 639–651. DOI: 10.1038/nprot.2013.024.

(5) Mirdita, M.; Schütze, K.; Moriwaki, Y.; Heo, L.; Ovchinnikov, S.; Steinegger, M. ColabFold: Making Protein Folding Accessible to All. *Nat. Methods.* **2022**, *19* (6), 679–682. DOI: 10.1038/s41592-022-01488-1.

(6) Jumper, J.; Evans, R.; Pritzel, A.; Green, T.; Figurnov, M.; Ronneberger, O.; Tunyasuvunakool, K.; Bates, R.; Žídek, A.; Potapenko, A.; Bridgland, A.; Meyer, C.; Kohl, S. A. A.; Ballard, A. J.; Cowie, A.; Romera-Paredes, B.; Nikolov, S.; Jain, R.; Adler, J.; Back, T.; Petersen, S.; Reiman, D.; Clancy, E.; Zielinski, M.; Steinegger, M.; Pacholska, M.; Berghammer, T.; Bodenstein, S.; Silver, D.; Vinyals, O.; Senior, A. W.; Kavukcuoglu, K.; Kohli, P.; Hassabis, D. Highly Accurate Protein Structure Prediction with AlphaFold. *Nature* **2021**, *596* (7873), 583–589. DOI: 10.1038/s41586-021-03819-2.

(7) Kroon, P. C.; Grunewald, F.; Barnoud, J.; van Tilburg, M.; Souza, P. C. T.; Wassenaar, T. A.; Marrink, S. J. Martinize2 and Vermouth: Unified Framework for Topology Generation. *Elife* **2023**, *12,* RP90627. DOI: 10.7554/eLife.90627.1.

(8) De Jong, D. H.; Singh, G.; Bennett, W. F. D.; Arnarez, C.; Wassenaar, T. A.; Schäfer, L. V.; Periole, X.; Tieleman, D. P.; Marrink, S. J. Improved Parameters for the Martini Coarse-Grained Protein Force Field. *J. Chem. Theory. Comput.* **2013**, *9* (1), 687–697. DOI: 10.1021/ct300646g.

(9) Periole, X.; Cavalli, M.; Marrink, S. J.; Ceruso, M. A. Combining an Elastic Network with a Coarse-Grained Molecular Force Field: Structure, Dynamics, and Intermolecular Recognition. *J. Chem. Theory. Comput.* **2009**, *5* (9), 2531–2543. DOI: 10.1021/ct9002114.

(10) Lomize, A. L.; Todd, S. C.; Pogozheva, I. D. Spatial Arrangement of Proteins in Planar and Curved Membranes by PPM 3.0. *Prot. Science.* **2022**, *31* (1), 209-220. DOI: 10.1002/pro.4219.
(11) Wassenaar, T. A.; Ingólfsson, H. I.; Böckmann, R. A.; Tieleman, D. P.; Marrink, S. J. Computational Lipidomics with Insane: A Versatile Tool for Generating Custom Membranes for Molecular Simulations. *J. Chem. Theory. Comput.* **2015**, *11* (5), 2144–2155. DOI: 10.1021/acs.jctc.5b00209.
(12) Souza, P. C. T.; Alessandri, R.; Barnoud, J.; Thallmair, S.; Faustino, I.; Grünewald, F.; Patmanidis, I.; Abdizadeh, H.; Bruininks, B. M. H.; Wassenaar, T. A.; Kroon, P. C.; Melcr, J.; Nieto, V.; Corradi, V.; Khan, H. M.; Domański, J.; Javanainen, M.; Martinez-Seara, H.; Reuter, N.; Best, R. B.; Vattulainen, I.; Monticelli, L.; Periole, X.; Tieleman, D. P.; de Vries, A. H.; Marrink, S. J. Martini 3: A General Purpose Force Field for Coarse-Grained Molecular Dynamics. *Nat. Methods.* **2021**, *18* (4), 382–388. DOI: 10.1038/s41592-021-01098-3.
(13) Bussi, G.; Donadio, D.; Parrinello, M. Canonical Sampling through Velocity Rescaling. J. Chem. Phys. **2007**, *126* (1), 014101. DOI: 10.1063/1.2408420.
(14) Parrinello, M.; Rahman, A. Polymorphic Transitions in Single Crystals: A New Molecular Dynamics *Method. J. Appl. Phys.* **1981**, *52* (12), 7182–7190. DOI: 10.1063/1.328693.
(15) Abraham, M. J.; Murtola, T.; Schulz, R.; Páll, S.; Smith, J. C.; Hess, B.; Lindah, E. Gromacs: High Performance Molecular Simulations through Multi-Level Parallelism from Laptops to Supercomputers. *SoftwareX* **2015**, *1,* 19-25. DOI: 10.1016/j.softx.2015.06.001.
(16) Humphrey, W.; Dalke, A.; Schulten, K. VMD: Visual Molecular Dynamics. *J. Mol. Graph.* **1996**, *14* (1), 33-38. DOI: 10.1016/0263-7855(96)00018-5.
(17) Song, W.; Yen, H. Y.; Robinson, C. V.; Sansom, M. S. P. State-Dependent Lipid Interactions with the A2a Receptor Revealed by MD Simulations Using In Vivo-Mimetic Membranes. *Structure* **2019**, *27* (2), 392-403. DOI: 10.1016/j.str.2018.10.024.
(18) Lee, J.; Patel, D. S.; Ståhle, J.; Park, S. J.; Kern, N. R.; Kim, S.; Lee, J.; Cheng, X.; Valvano, M. A.; Holst, O.; Knirel, Y. A.; Qi, Y.; Jo, S.; Klauda, J. B.; Widmalm, G. & Im, W. CHARMM-GUI Membrane Builder for Complex Biological Membrane Simulations with Glycolipids and Lipoglycans. *J. Chem. Theory. Comput.* **2019**, *15*, 775–786. DOI: 10.1021/acs.jctc.8b01066.
(19) Huang, J.; Rauscher, S.; Nawrocki, G.; Ran, T.; Feig, M.; De Groot, B. L.; Grubmüller, H. & MacKerell, A. D. CHARMM36m: An improved force field for folded and intrinsically disordered proteins. *Nat. Methods.* **2016**, *14* (2), 71-73 DOI: 10.1038/nmeth.4067
(20) Jorgensen, W. L.; Chandrasekhar, J.; Madura, J. D.; Impey, R. W. & Klein, M. L. Comparison of simple potential functions for simulating liquid water. *J. Chem. Phys.* **1983**, *79*, 926–935. DOI: 10.1063/1.445869
(21) Evans, D. J. & Holian, B. L. The Nose-Hoover thermostat. *J. Chem. Phys.* **1985**, *83*, 4069–4074. DOI: 10.1063/1.449071
(22) Mori, K., Matsumoto, N., Nomoto, S. & Tsuruta, K. Computational and Experimental Analyses of Detachment Force at the Interface between Carbon Fibers and Epoxy Resin. *Proc. Comput. Mech. Conf.* **2017**, *30*, 196. DOI: 10.4236/ojcm.2017.74011
(23) Petersen, H. G. Accuracy and efficiency of the particle mesh Ewald method. *J. Chem. Phys.* **1995***, 103*, 3668–3679. DOI: 10.1063/1.470043
(24) Hess, B., Bekker, H., Berendsen, H. J. C. & Fraaije, J. G. E. M. LINCS: A Linear Constraint Solver for molecular simulations. *J. Comput. Chem*. **1997**, *18*, 1463–1472. DOI: 10.1002/(SICI)1096-987X(199709)18:123.0.CO;2-H
